# Hexacoordinate Ru-based olefin metathesis catalysts with pH-responsive N-heterocyclic carbene (NHC) and N-donor ligands for ROMP reactions in non-aqueous, aqueous and emulsion conditions

**DOI:** 10.3762/bjoc.11.212

**Published:** 2015-10-21

**Authors:** Shawna L Balof, K Owen Nix, Matthew S Olliff, Sarah E Roessler, Arpita Saha, Kevin B Müller, Ulrich Behrens, Edward J Valente, Hans-Jörg Schanz

**Affiliations:** 1 Department of Chemistry & Biochemistry, The University of Southern Mississippi, 118 College Drive, Hattiesburg, MS 39406-5043, USA; 2 Department of Chemistry, Georgia Southern University, 521 College of Education Drive, Statesboro, GA 30458-8064, USA; 3 BASF SE, G-PM/PD - F206, 67056 Ludwigshafen, Germany; 4 BASF SE, Basic Chemicals Research, GCB/C – M313, 67056 Ludwigshafen, Germany; 5 Department of Chemistry, University of Portland, 5000 N. Willamette Blvd., Portland, OR 97203, USA

**Keywords:** activation, aqueous catalysis, emulsion, olefin metathesis, polymerization, ruthenium

## Abstract

Three new ruthenium alkylidene complexes (PCy_3_)Cl_2_(H_2_ITap)Ru=CHSPh (**9**), (DMAP)_2_Cl_2_(H_2_ITap)Ru=CHPh (**11**) and (DMAP)_2_Cl_2_(H_2_ITap)Ru=CHSPh (**12**) have been synthesized bearing the pH-responsive H_2_ITap ligand (H_2_ITap = 1,3-bis(2’,6’-dimethyl-4’-dimethylaminophenyl)-4,5-dihydroimidazol-2-ylidene). Catalysts **11** and **12** are additionally ligated by two pH-responsive DMAP ligands. The crystal structure was solved for complex **12** by X-ray diffraction. In organic, neutral solution, the catalysts are capable of performing standard ring-opening metathesis polymerization (ROMP) and ring closing metathesis (RCM) reactions with standard substrates. The ROMP with complex **11** is accelerated in the presence of two equiv of H_3_PO_4_, but is reduced as soon as the acid amount increased. The metathesis of phenylthiomethylidene catalysts **9** and **12** is sluggish at room temperature, but their ROMP can be dramatically accelerated at 60 °C. Complexes **11** and **12** are soluble in aqueous acid. They display the ability to perform RCM of diallylmalonic acid (DAMA), however, their conversions are very low amounting only to few turnovers before decomposition. However, both catalysts exhibit outstanding performance in the ROMP of dicyclopentadiene (DCPD) and mixtures of DCPD with cyclooctene (COE) in acidic aqueous microemulsion. With loadings as low as 180 ppm, the catalysts afforded mostly quantitative conversions of these monomers while maintaining the size and shape of the droplets throughout the polymerization process. Furthermore, the coagulate content for all experiments stayed <2%. This represents an unprecedented efficiency in emulsion ROMP based on hydrophilic ruthenium alkylidene complexes.

## Introduction

The vast application spectrum of Ru-based olefin metathesis has provided a powerful synthetic tool for the organic [1–3] and polymer chemist [4–8] alike. The catalysts’ high tolerance towards functional groups, air and moisture makes them attractive to be used in combination of a wide range of substrates and solvents [9–12]. Over the past decade, Ru–alkylidene based olefin metathesis in aqueous media has become increasingly important [13]. Benefits such as the non-hazardous, vastly abundant and commercially highly attractive of water coupled with a high heat capacity make organic transformations using hydrophilic catalyst in aqueous media very attractive [14–18]. These benefits, coupled with potential applications in biological media [19], have led to the development of various water-soluble catalyst designs [20–21]. Such catalysts contain hydrophilic phosphine ligands [22–25], NHC ligands [26–29], N-donor ligands [30], alkylidene moieties [31–33] or combinations of these structural features [34–37]. Another recent development in homogeneous catalysis, and olefin metathesis in particular, have become switchable catalysts or systems where the activity can be controlled by external stimuli [38–44]. In metathesis, pH is a very straightforward stimulus that can fulfill two independent functions for catalysts bearing pH-responsive ligands resulting in metathesis activation [45–53] and/or solubilization [31–32] in aqueous media.

**Figure 1:**
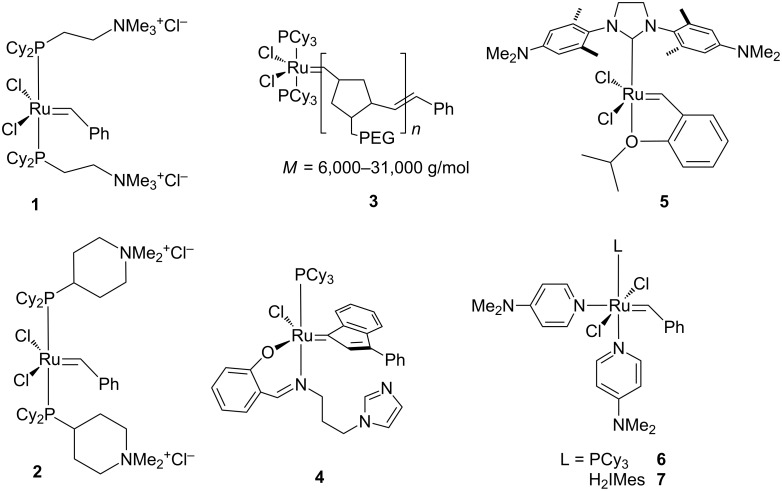
Hydrophilic and/or pH-responsive Ru–alkylidene complexes **1–7** for olefin metathesis.

One of the most intriguing applications of water-soluble metathesis catalysts is the production of latexes via ring-opening metathesis polymerization (ROMP) in emulsion. However, to date very few reports have successfully employed well-defined, hydrophilic Ru–alkylidene catalysts in combination with a hydrophobic monomer in emulsion. The first emulsion ROMP was reported in the early 2000’s when Claverie et al. used 1^st^ generation Grubbs-type catalysts [24] **1** and **2** (Figure 1, approx. 400 ppm catalyst loading) to effectively polymerize norbornene (NBE) at 80 °C in microemulsion (91% conversion) [54]. The same conditions failed to polymerize significant amounts of the far less reactive monomers cyclooctene (COE) or cyclooctadiene (COD) with yields below 10%. Later, Heroguez et al. synthesized the 1^st^ generation Grubbs-type macroinitiator **3** which accomplished near quantitative conversions with NBE and as high as 90% conversion with COE and COD using 500 ppm catalyst loading in microemulsion [55]. However, these high conversions were accompanied by flocculation of the polymers. Just recently, Maier et al. reported pH-responsive catalyst **4** which accomplished up to 95% ROMP conversion with 0.2% catalyst loading in microemulsion after the addition of HCl [56]. Interestingly, increased acid addition resulted in an increased molecular weight control of the emulsion ROMP process. To date, no hydrophilic catalyst has been reported to be employed for emulsion ROMP bearing an NHC ligand. This may a consequence of the low accessibility of these catalysts and one of the reasons for the relatively low observed activities knowing that the NHC ligand dramatically increases the propagation rates of the metathesis reaction [57]. The higher accessibility of water-insoluble catalysts has resulted in an increased investigation of water-insoluble Ru–alkylidene complexes for emulsion ROMP in aqueous media. Slugovc et al. reported the ROMP of dicyclopentadiene (DCPD) in a “high internal phase emulsion” (HIPE) of the monomer in water [58]. Stable latexes have been produced by use of organic-soluble catalysts in micro or miniemulsions [59–60]. Although, this technique has been more successfully applied for a variety of ROMP substrates and allowed the use of more metathesis-active NHC-bearing catalysts, the protocols required to emulsify the catalyst and monomer separately in significant amounts of an organic cosolvent. From a practical and environmental standpoint, the use of hydrophilic complexes for emulsion ROMP eliminating or reducing the need for high amounts of organic cosolvents seems advantageous. In this light it is desirable to develop water-soluble catalyst systems which can perform the task with high activity, substrate range and sufficient hydrolytic stability to access a variety of novel ROMP latexes. We now wish to report the synthesis of two new pH-responsive, Ru-based olefin metathesis catalysts, their ROMP and ring closing metathesis (RCM) activities in organic and aqueous solvents, as well as their use in the first near-quantitative ROMP procedure in microemulsion to produce stable latexes from DCPD and DCPD/COE mixtures.

## Results and Discussion

### Catalyst syntheses

We have previously reported olefin metathesis catalyst **5** bearing the pH-responsive H_2_ITap [1,3-bis(*N*’,*N*’,2’,6’-tetramethylaminophenyl)-4,5-dihydroimidazol-2-ylidene] ligand containing two NMe_2_ groups [61]. The addition of HCl to complex **5** results in the protonation of the amino groups to produce a water-soluble dicationic complex. Although the protonation of the ancillary NMe_2_ groups was demonstrated to cause a reduced ROMP propagation rate compared the neutral catalyst [62], we hypothesized that a catalyst based on this NHC-motif could still be superior in activity to phosphine-containing catalysts **1–4** in an emulsion ROMP process. It should be noted that olefin metathesis catalysts bearing a similar ligand with NEt_2_ groups instead of the NMe_2_ groups of the H_2_ITap ligand have been developed simultaneously in Plenio’s laboratories [63].

**Scheme 1:**
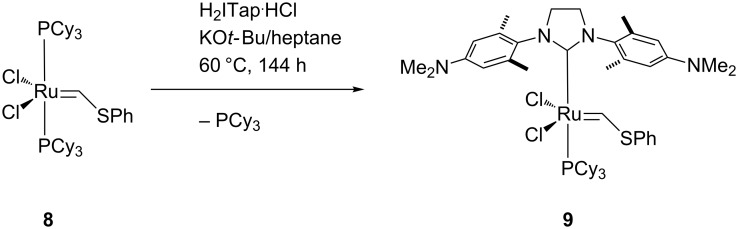
Synthesis of 2^nd^ Grubbs-type generation complex **9**.

**Scheme 2:**
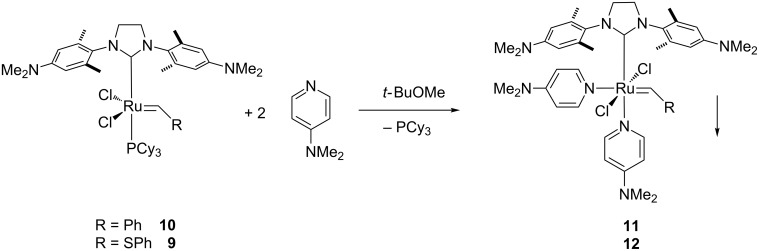
Synthesis of hexacoordinate, pH-responsive complexes **11** and **12**.

We anticipated that a variety of Ru-based olefin metathesis catalysts with an H_2_ITap ligand should be accessible quite straightforwardly to be used in emulsion ROMP. For this purpose, we synthesized 2^nd^ generation Grubbs-type catalyst **9** from ruthenium phenylthiomethylidene complex **8** in a modified ligand exchange procedure (Scheme 1), which is somewhat analogous to the most common literature procedure [61,64]. The ROMP and RCM performance of Fischer-carbene complexes such as **9** are often sluggish and often do not result in high conversions [65–66]. However, these complexes are thermally very inert and economically viable options to other commercially available olefin metathesis catalyst. Furthermore, their use at elevated temperatures may be feasible or even advantageous over their more reactive counterparts. Since catalyst **9** is not very soluble in aqueous HCl despite double protonation we replaced the hydrophobic PCy_3_ ligand with two 4-dimethylaminopyridine (DMAP) ligands. This was demonstrated to significantly improve the complex solubility in acidic aqueous media [32]. We have also demonstrated before that acid addition to (DMAP)_2_Ru–alkylidene complexes **6** and **7** resulted in fast protonation of the N-donor ligand and thus resulting in fast, irreversible and generally complete metathesis initiation [45–46]. For most ROMP processes, this is desirable as a fast initiation typically affords high ROMP activity with low catalyst loadings [57,67]. Hence, hexacoordinate complexes **11** and **12** were also synthesized from their precursor complexes **9** and **10** [61] by ligand exchange according to Scheme 2. These complexes now contain pH-responsive groups at the H_2_ITap ligand to afford solubility in aqueous acid and at the N-donor ligand which affords rapid metathesis initiation. It should be noted that Plenio et al. also reported a Ru–benzylidene complex similar to catalysts **11** and **12** bearing the NEt_2_-analogue to the H_2_ITap ligand and two pyridine ligands instead of DMAP. The pH-responsive nature of this complex caused a change in the *E*/*Z*-selectivities of ROMP reactions upon acid addition but the catalyst was not tested for aqueous or emulsion ROMP [68].

### Crystal structure analysis of complex **12**

**Figure 2:**
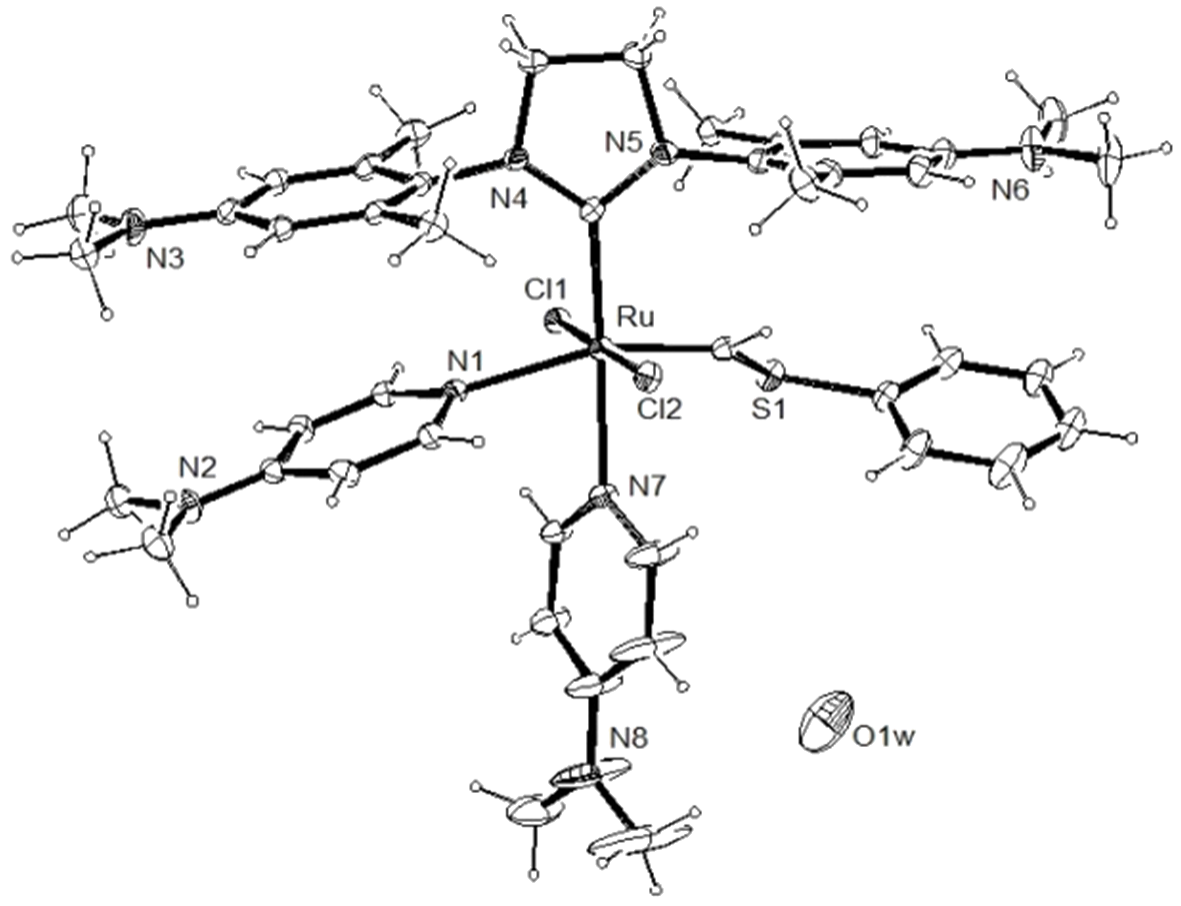
ORTEP diagram for H_2_ITap(DMAP)_2_Cl_2_Ru=CH-SPh (**12**). The positions of the hydrogen atoms were calculated. The unit cell contains a molecule of cocrystallized water. The hydrogen atoms of the water molecule were omitted from the structure due to thermal uncertainty.

Crystals of complex **12** suitable for X-ray diffraction were obtained via layer diffusion of heptane into a concentrated THF solution (Figure 2). Hexacoordinated complex **12** adopts the expected distorted octahedral coordination sphere around the Ru center with *trans* chloride and *cis* DMAP ligands. In comparison to complex **13** [46], the only other (DMAP)_2_Cl_2_Ru–alkylidene complex bearing an NHC ligand for which a crystal structure was solved, all metal ligand bond distances are very similar (within 2 pm) with the exception of one Ru–N distance to the DMAP ligand *trans* to the NHC ligand (Table 1). In complex **12** this distance is shorter by >0.04 Å. This may be a result of the bridging S-atom in the alkylidene moiety which increases the distance of the phenyl ring to the metal center and the surrounding ligands. Hence, a reduced steric repulsion of this phenyl ring on the geometry around the metal could result, in particular the sterically close N-donor ligand. This can also be seen in the cis C=Ru–N angle which is smaller by almost 2° allowing a closer proximity of these two moieties.

**Table 1:** Selected bond lengths (Å) and angles (°) for complexes **12** and **13** [46].

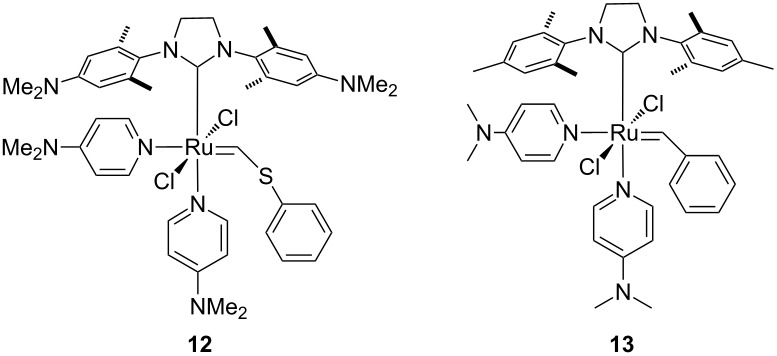					

	**12**	**13**		**12**	**13**

Ru=C	1.874(5)	1.873(2)	Ru–C	2.057(4)	2.051(2)
Ru–N	2.201(4) 2.289(4)	2.1933(16) 2.3309(17)	Ru–Cl	2.4091(11) 2.4202(11)	2.3847(5) 2.4372(5)

C=Ru–C	96.22(17)	95.00(9)	Cl–Ru–Cl	179.25(4)	177.54(2)
C=Ru–N	176.86(13) 86.32(12)	176.64(7) 88.29(8)	C–Ru–N	163.28(15) 99.66(15)	162.41(8) 97.01(7)
C=Ru–Cl	93.02(14) 86.33(12)	90.47(6) 85.43(7)	C–Ru–Cl	92.42(12) 87.58(12)	88.29(8) 89.07(5)

### Catalytic experiments

We investigated the catalyst activity of novel complexes **9**, **11** and **12** in the ROMP of cyclooctene (COE, [Ru] = 0.5 mM, 0.5 mol % catalyst loading) and the ring-closing metathesis (RCM) of diethyl diallylmalonate (DEDAM, [Ru] = 1.0 mM, 1% (n/n) catalyst loading) in neutral organic media (Table 2). The ROMP reaction with catalyst **11** in benzene-*d*_6_ accomplished 93% conversion of COE within 19 min which is similar in the performance to its previously reported counterpart **13**. Interestingly, the same reaction is accelerated and yields near quantitative (97%) conversion in 15 min in the presence of 2 equiv of H_3_PO_4_ as a result of the protonation of the DMAP ligands and hence, fast and complete initiation. The addition of more acid (4 equiv H_3_PO_4_) results in a reduction of the activity (41% in 30 min). This may be due to significant protonation of the H_2_ITap ligand which was shown to have an adverse effect on the metathesis propagation of these complexes [61–62]. By contrast, complex **12** exhibited much lower activity as expected. The ROMP of COE in CDCl_3_ at ambient temperature only affords 3.9% conversion in 60 min. CDCl_3_ was used owing to the low complex solubility in benzene-*d*_6_ and it should be noted that complex **12** is stable for several hours at ambient temperature (<2% decomposition in 2 h) in this solvent. Heating the reaction to 60 °C increases the catalyst activity (36% conversion in 60 min), however, the reaction does not reach completion likely owing to catalyst degradation. In contrast to complex **11**, the addition of 2 equiv of acid proved counter-effective for complex **12** (0.9% conversion in 60 min). It appears that the fast and complete dissociation of the DMAP ligand with this catalyst is not synonymous with the metathesis initiation. This means, while an activated species is formed, other processes, including decomposition are faster than metathesis resulting in minimal portion of complex **12** affording ROMP. 2^nd^ generation Grubbs-type catalyst **9** by contrast exhibited a pronounced acceleration in the ROMP of COE when heated. The reaction at ambient temperature did not afford noticeable amounts of product (<1% conversion) in 60 min, however, at 60 °C, the conversion reached 96% in less than half the time period. The low metathesis activity of Fischer-type Ru–alkylidenes at room temperature is well-documented [66]. The observed acceleration with heat indicates a significant latency for this complex based on slow metathesis initiation. Neither complexes **11** or **12** performed efficiently in the RCM of DEDAM due to rapid degradation of the catalyst. Whereas catalyst **11** levels off at 7.2% conversion after 30 min at room temperature, catalyst **12** needed to be heated to 60 °C to be activated, and no further conversion was monitored after 60 min (57%). It is likely that the observed low catalyst stability observed for the reactions with complex **11** in benzene solution is based on the rapid degradation of the corresponding (DMAP)_2_Ru=CH_2_ intermediate. Such a labile methylidene intermediate is not produced in the ROMP reactions making it the much more effective process. Catalyst **12** produces the very same intermediate, however, the RCM and ROMP reactions both exhibited rapid catalyst decomposition. It appears likely that other degradation mechanisms possibly influenced by the chlorinated solvent (CDCl_3_) are also involved. Therefore it was not surprising that DMAP-free complex **9** performed quite efficiently in the RCM of DEDAM, more so than complexes **11** and **12**. While at room temperature, the slow metathesis initiation of complex **9** limited the conversion rates dramatically (2% after 60 min), at 60 °C, 90% conversion of DEDAM were monitored in 60 min resulting in a performance much more similar to other 2^nd^ generation Grubbs-type catalysts [61,69].

**Table 2:** ROMP and RCM reactions with catalysts **8–10** in C_6_D_6_ ([Ru] = 0.5 mM for 0.5 mol %, 1.0 mM for 1 mol % loading).

catalyst	catalyst loading (%)	substrate	product	equiv H_3_PO_4_	time (min)	temperature (°C)	conversion (%)

**9**	0.5	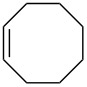 COE	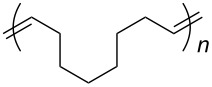	0	60	20	0.8
**9**				0	24	60	96
**11**				0	19	20	93
**11**				2	15	20	97
**11**				4	30	20	41^a^
**12**^b^				0	60	20	3.9
**12**^b^				0	30	60	32
**12**^b^				0	60	60	36^a^
**12**^b^				2	60	20	0.9^a^

**9**	1.0	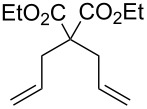 DEDAM	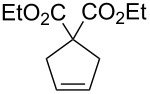	0	60	20	2.3
**9**				0	30	60	81
**11**				0	30	20	7.2
**11**				2	30	20	47^a^
**11**				4	30	20	14^a^
**12**^b^				0	60	20	1.2
**12**^b^				0	30	60	50
**12**^b^				0	180	60	61^a^

^a^No significant conversion after that time period due to catalyst precipitation or decomposition; ^b^in CDCl_3_.

In contrast to complex **9**, complexes **11** and **12** are completely soluble in aqueous acid. Similar to complex **5**, no noticeable aqueous ROMP was accomplished but the RCM of diallylmalonic acid (DAMA) afforded somewhat low conversions (Table 3) inferior to complex **5**. Based on the observed reactivity trend from the previous kinetic experiments, it is not surprising that benzylidene complex **11** exhibited a superior performance in aqueous HCl where complex **12** failed to produce noticeable amounts of ring-closed product. Interestingly however, when the aqueous solvent is changed to 0.1 m H_3_PO_4_, complex **12** exhibited a similar performance to catalyst **11**. In fact, this is the only time catalyst **12** exhibited an appreciable metathesis reaction in an acidic medium.

**Table 3:** RCM of diallylmalonic acid (DAMA) in 0.1 M aqueous acid ([Ru] = 2.0 mM, 4 mol % catalyst loading).

catalyst	substrate	product	acid	time (min)	temperature (°C)	conversion (%)

**5**^a^	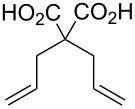 DAMA	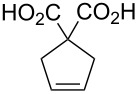	HCl	30	50	44^b^
**11**			HCl	30	50	25^b^
**11**			H_3_PO_4_	30	50	8.7
**12**			HCl	60	50	n.o.
**12**			H_3_PO_4_	60	50	10.3^b^

^a^See [61]; ^b^no further conversion after this time period.

### Emulsion ROMP

**Scheme 3:**
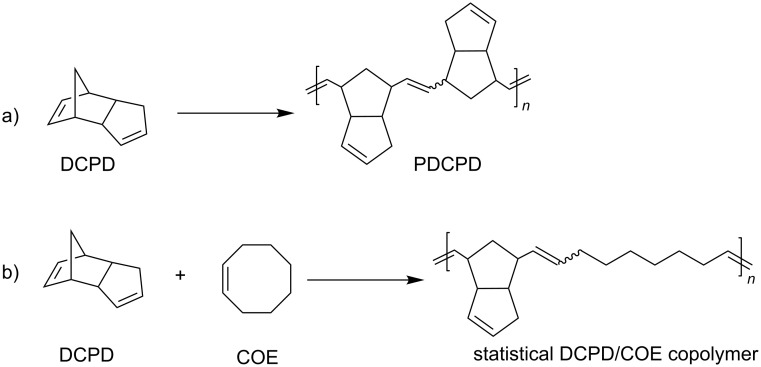
ROMP reactions conducted under microemulsion conditions.

Based on their solubility in aqueous acid, we were investigating the suitability of catalysts **11** and **12** for the ROMP of DCPD and a DCPD/COE mixture (49:51 mol/mol) in microemulsion to give polydicyclopentadiene (PDCPD) or a statistical copolymer of DCPD and COE (Scheme 3). A 0.1 M HCl_aq_ catalyst solution was added to an emulsion of the monomer containing *n*-hexadecane (5% by mass) to improve the monomer liquidity and polyethylene glycol (PEG) based Emulgin^®^ B3 as surfactant which was previously vigorously stirred for 1 h and then further emulsified using a sonication probe for another 5 min establishing the microemulsion. The emulsion polymerization reactions were conducted at less favorable conditions than those with all previous hydrophilic catalysts. The two different temperatures (35 °C and 55 °C or 65 °C) are significantly lower than 80 °C, which has been commonly used with previous hydrophilic catalysts [54–56]. Furthermore, DCPD and COE exhibit a much lower ROMP activity than NBE, the monomer of choice for previous applications. Finally, catalyst loadings of 180–200 ppm were used which is the lowest reported thus far for any emulsion ROMP reaction. With exception of ROMP of DCPD/COE with catalyst **12** at 35 °C, all reactions proceeded to near-quantitative degree as their determined solid contents often times exceeded the theoretical value derived from the amounts of monomer and surfactant added. This indicates that the catalysts have sufficient activity and thermal stability under the chosen conditions to promote complete ROMP of DCPD and the DCPD/COE monomer mixture.

After the reaction, the latex was filtered (20 mm filter) and the coagulated contents were determined. The z-average droplet diameter was measured via autosizer and a small sample was removed to determine the solid content in the moisture meter. The obtained latexes were relatively stable and could be stored without flocculation. Most reactions provided levels of <1% coagulate versus the dispersed polymer in the latex. In fact, catalyst **12** at 35 °C produced very low levels of coagulum (0.1%) for both reactions. At the higher temperatures, the coagulation increased but the levels always stayed <2%. The average latex particle diameters range from 255 nm to 315 nm using the same concentration of surfactant throughout the series of experiments. The final average droplet diameter deviated less than 3% from the initial droplet size before polymerization where determined. Therefore, the size of the latex particles is somewhat controllable. It should be noted that DCPD contains two reactive double bonds in the monomer structure. When both undergo metathesis in a ROMP reaction, particularly at elevated temperatures, then the PDCPD material is crosslinked [70]. With respect to the latexes synthesized in this project, the presence or the degree of crosslinking in the material has not been determined. The results of the emulsion ROMP experiments are summarized in Table 4.

**Table 4:** Emulsion ROMP of DCPD (Ru/monomer = 1:5.0 × 10^4^) and DCPD/COE (49:51 (mol/mol) – Ru/monomer = 1:5.6 × 10^4^) mixtures with catalysts **11** and **12** after 120 min reaction time.

catalyst (in 0.012 M HCl)	temperature (°C)	monomer	catalyst loading (ppm)	conversion^a^ (%)	coagulate (%)	av. particle diameter (nm)

**11**	35	DCPD	200	>99	0.4	269
**11**	55	DCPD		>99	1.0	278
**12**	35	DCPD		99	0.1	315
**12**	65	DCPD		>99	0.9	265
**11**	35	DCPD/COE 1:1	180	>99	0.4	270
**11**	65	DCPD/COE 1:1		>99	1.5	264
**12**	35	DCPD/COE 1:1		92	0.1	255
**12**	65	DCPD/COE 1:1		>99	1.6	290

^a^Conversion determined by weight analysis of non-volatile material left after drying.

**Scheme 4:**
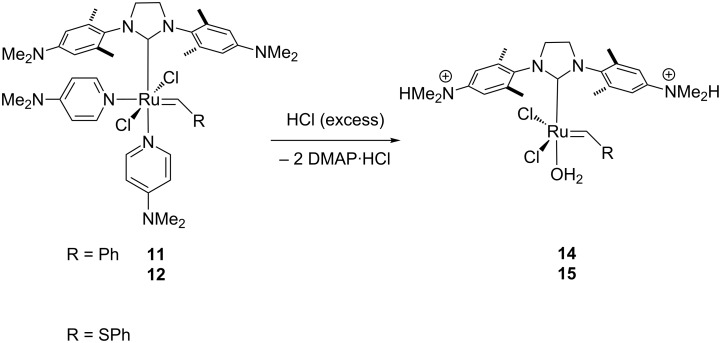
Proposed formation of catalytic species **14** and **15** under emulsion ROMP conditions.

Evidently, NHC-ligated catalysts **11** and **12** exhibit a much elevated activity under microemulsion conditions in comparison to their water-soluble predecessors **1–3** [8,11–12]. At first glance, these high turnover numbers are in stark contrast to the observed low metathesis activity of catalysts **11** and particularly **12** in homogeneous acidic aqueous solution. Based on the low catalyst loadings used in the experiments, their metathesis activity appears to be increased by several orders of magnitude by comparison, meaning the reaction environment must have changed from aqueous to organic. This means, the ROMP reaction is most likely occurring within the micelles. About the nature of the catalytic Ru species can only be speculated at this point. It seems likely that the aqueous acid has completely protonated the pH-responsive ligands to produce water-soluble complexes **14** and **15** (Scheme 4). The protonation of the H_2_ITap ligand with aqueous DCl has been demonstrated to be effective, if not quantitative, for complex **5** [61]. The partial or complete removal of donor ligands from Ru–alkylidene complexes with strong aqueous acids has also been shown before which then resulted in catalytic species with higher metathesis activity [23,45–46]. In these cases, the empty coordination site was proposed to be occupied by a weak O-donor ligand, i.e., a water molecule which also resulted in a significant stabilization of these activated species from thermal degradation. Since lowering the degree of protonation in H_2_ITap ligated Ru–alkylidene complexes has been demonstrated to improve the catalytic activity [62], it cannot be ruled out that the ROMP active species in the micelle may be partially or even completely deprotonated. Also, in the micelle, the H_2_O concentration is significantly reduced which could be another reason that a solvent-based inhibition as observed in aqueous media is minimal at best. With regard to the stability of Ru–alkylidene complexes **14** and **15**, they should exhibit much lower thermal stability due to high initiation rates [57]. However, the ability to quantitatively convert the monomers indicates that species **14** and **15** either are stabilized in the aqueous solvent, i.e., via H_2_O donation, or the species rapidly migrate into the monomer droplets where they are protected by the monomer as seen previously [58].

**Figure 3:**
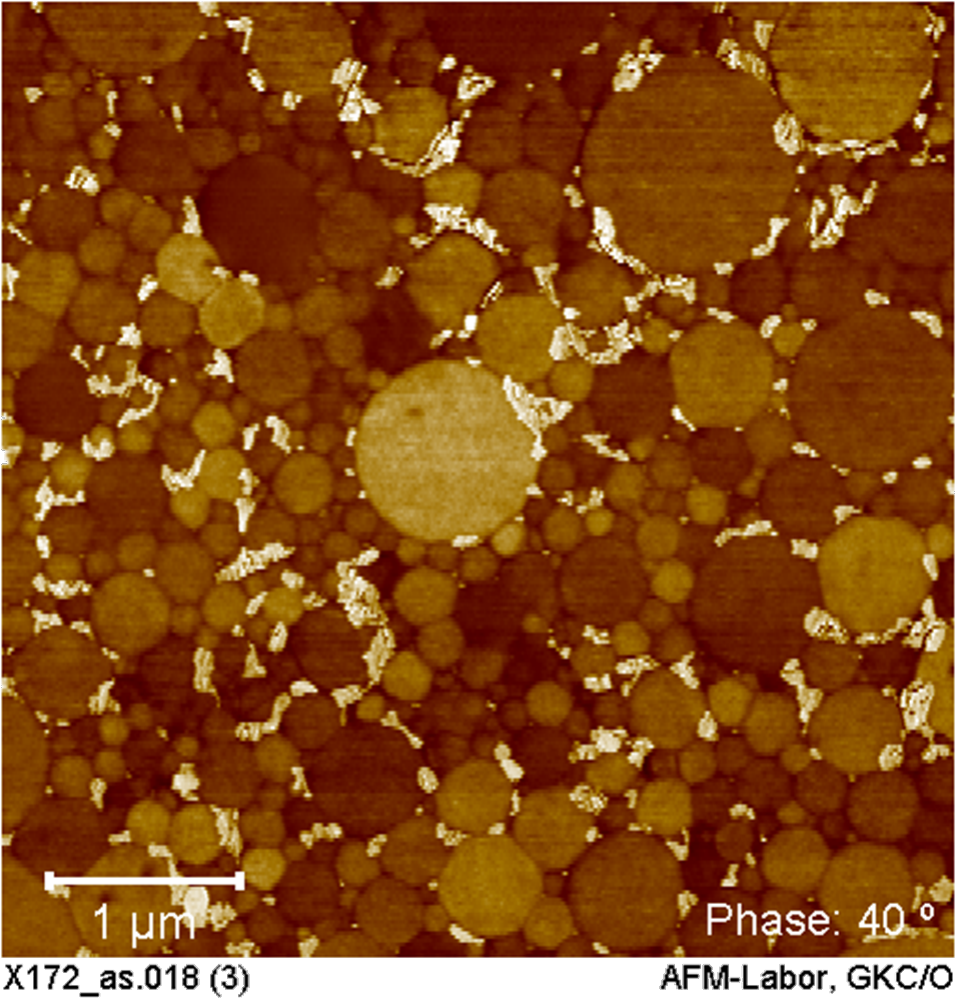
AFM image produced from COE/DCPD latex film. Measurement: AFM tapping at room temperature, material contrast using Phase Imaging.

A film was produced from the COE/DCPD latex from the ROMP reaction with catalyst **12** at 65 °C. The film was dried at room temperature and cut using a Cryo-Microtome. After the procedure, the spherical particles maintain their size and shape in the film as shown in the atom force microscope (AFM) image (Figure 3).

## Conclusion

In conclusion, the three new olefin metathesis catalysts **9**, **11** and **12** bearing the pH-responsive H_2_ITap ligand were synthesized and tested in RCM and ROMP reactions. Complex **12** was characterized via X-ray diffraction. While in homogeneous organic or aqueous solution, the 2^nd^ generation Grubbs-type catalyst **9** containing a Fischer-type phenylthiomethylidene group exhibited significant latency but proved to be a proficient ROMP and RCM catalyst at elevated temperatures. Catalyst **11** exhibited the typical high ROMP activity for a third-generation Grubbs-type catalyst in benzene. The ROMP reaction could even be strongly accelerated when two equivalents of a strong acid were added to the catalyst. However, in RCM reactions or in acidic aqueous media, catalyst **11** suffered from rapid degradation. By contrast, catalysts **12** exhibited relatively low conversions for all metathesis reactions in homogeneous solution due to slow metathesis initiation and/or rapid catalyst degradation. However, both catalysts **11** and **12** proved to be extremely capable of ROMP in microemulsion of DCPD and COE. The (co)polymers were formed in near-quantitative yields with catalyst loadings as low as 180 ppm while forming stable latexes with minimal coagulation (0.1–1.6%). The latex particles maintain their size (between 255 and 315 nm) and shape throughout the polymerization and the processing into the film. This is the first time that hydrophilic, NHC-ligated olefin metathesis catalysts were used in emulsion ROMP. Catalysts **11** and **12** demonstrated a superior ability for this process by using the lowest ever catalyst loading for two monomers with significantly lower ROMP activity than the typically used NBE monomer at moderate temperatures while routinely affording near-quantitative conversions. Further investigations of the emulsion ROMP process with respect to the nature of catalytic species in the micelle and the properties of the resulting latexes and materials are currently under way.

## Experimental

### General procedures

All experiments with organometallic compounds were performed under a dry nitrogen atmosphere using standard Schlenk techniques or in an MBraun drybox (O_2_ < 2 ppm). NMR spectra were recorded with a Varian Inova instrument (300.1 MHz for ^1^H, 75.9 MHz for ^13^C, and 121.4 MHz for ^31^P) and an Agilent 400 MHz MR system (400.0 MHz for ^1^H, 100.6 MHz for ^13^C, and 162.9 MHz for ^31^P). ^1^H and ^13^C NMR spectra were referenced to the residual solvent, ^31^P NMR spectra were referenced using H_3_PO_4_ (δ = 0 ppm) as external standard. The crystallographic properties and data were collected using Mo Kα radiation and the charge-coupled area detector (CCD) detector on an Oxford Diffraction Systems Gemini S diffractometer. The solid contents of latexes were determined using a Mettler Toledo HR73 moisture meter. The droplet diameter was determined using an Autosizer IIC from Malvern Instruments.

**Materials and methods. ***n-*Heptane, THF, CH_2_Cl_2_ and *t-*BuOMe were dried by passage through solvent purification (MBraun-Auto-SPS). C_6_D_6_ and CDCl_3_ were degassed prior to use. 2-PrOH was used without further purification. Complex **8** was donated by BASF SE and used as delivered. Other chemicals and reagents were purchased from commercial sources, and they were degassed and stored in the dry-box when directly used in combination with organometallic complexes, and otherwise were used without further purification. H_2_ITap∙HCl, complex **8**, as well as DEDAM and DAMA were synthesized according to literature procedures [61,71].

**Synthesis of (1,3-bis(2’,6’-dimethyl-4’-dimethylaminophenyl)-2-dihydroimidazolidinylidene)dichloro(phenylthiomethylene)(tricyclohexylphosphine)ruthenium(II) (PCy_3_)Cl_2_(H_2_ITap)Ru=CHSPh (9):** H_2_ITap∙HCl (567 mg, 1.41 mmol) and KO*t-*Bu (180 mg, 1.61 mmol) were heated to 80 °C in heptane (120 mL) for 90 min. After the slurry cooled to room temperature, (PCy_3_)_2_Cl_2_Ru=CHSPh (**8**, 969 mg, 1.13 mmol) was added and the mixture was stirred at 60 °C for 144 h. The solvent was then removed under reduced pressure and 2-PrOH/water (3:1 v/v) was added (70 mL) and the slurry was sonicated at 30 °C for 60 min. The mixture was filtered in air, the residue was washed once with 2-PrOH (20 mL), and then the residue was dried in the vacuum oven at 60 °C for 4 h. The residue still contained significant amounts of the starting complex (on average approx. 30%). Cyclohexane (80 mL) was added to the dry residue (666 mg) under inert gas and sonicated at 30 °C for 60 min. The slurry was filtered in air, the residue was washed with cyclohexane (2 × 15 mL) and then dried in the vacuum oven at 60 °C for 2 h to give compound **9** (378 mg, 0.40 mmol, 36%) in >99% purity. ^1^H NMR (300.1 MHz, C_6_D_6_, 20 °C) δ 17.99 (s, Ru=*CH*), 7.23 (d, ^3^*J*[^1^H^1^H] = 7.2 Hz, 2H), 6.97 (t, ^3^*J*[^1^H^1^H] = 8.4 Hz, 1H), 6.89 (m, 2H, =CH-C_6_*H*_5_), 6.51 (s, 2H), 6.14 (s, 2H, 2 × C_6_*H*_2_), 3.36 (m, 4H, C*H*_2_-C*H*_2_), 2.90 (s, 6H), 2.76 (s, 6H, 2 × N(C*H*_3_)_2_), 2.61 (s, 6H), 2.29 (s, 6H, 2 × C_6_H_2_(C*H*_3_)_2_), 2.57 (br, m, 3H), 1.88 (br, m, 6H), 1.65 (br, m, 6H), 1.55 (br, m, 3H), 1.45–1.02 (br, m, 18H, PCy_3_); ^13^C {^1^H} NMR (75.9 MHz, C_6_D_6_, 20 °C) δ 272.5 (br, Ru=*C*H), 219.7 (d, ^2^*J*[^31^P^13^C] = 81.1 Hz, N-*C*-N), 150.9, 149.9, 142.2, 140.8, 139.0, 129.7, 129.0, 126.8, 125.9, 125.8, 113.0, 112.3 (s, aryl-*C*), 52.7, 52.5 (s, N-*C*H_2_-*C*H_2_-N), 40.4, 40.0 (N-*C*H_3_), 21.4, 20.4 (C_6_H_2_(*C*H_3_)_2_), 32.8 (d, ^1^*J*[^31^P^13^C] = 14.9 Hz), 30.0 (s), 28.5 (d, ^2^*J*[^31^P^13^C] = 10.1 Hz), 27.2 (s, PCy_3_); ^31^P {^1^H} NMR (121.4 MHz, C_6_D_6_, 20 °C) δ 23.4 (s); Anal. calcd for C_44_H_58_Cl_2_N_8_Ru: C, 60.68; H, 6.71; N, 12.87; found: C, 60.21; H, 6.77, N, 12.27.

**Recovery of bis(tricyclohexylphosphine)dichloro(phenylthiomethylene)ruthenium(II) (PCy_3_)_2_Cl_2_Ru=CHSPh (8).** The cyclohexane filtrate and washes were combined and dried under reduced pressure. Acetone (30 mL) was added to the remaining solid and the slurry was sonicated for 30 min at 30 °C. The mixture is filtered in air and the residue was washed with acetone (2 × 15 mL). Then the filter residue was dried in the vacuum oven at 60 °C for 2 h to recover 301 mg of material (approx. 31%). The ^1^H NMR analysis showed that the residue was only composed of compound **8** (96%) and compound **9** (4%). The recovered catalyst was mixed with **9** in later synthesis reactions to synthesize **9**.

**Synthesis of benzylidene(1,3-bis(2’,6’-dimethyl-4’-dimethylaminophenyl)-2-dihydroimidazolidinylidene)bis(4-dimethylaminopyridine)dichlororuthenium(II) (DMAP)_2_Cl_2_(H_2_ITap)Ru=CHPh (11):** 4-Dimethylaminopyridine (DMAP, 315 mg, 2.58 mmol) was added to a slurry of (PCy_3_)Cl_2_(H_2_ITap)Ru=CHPh (**10**, 987 mg, 1.09 mmol) in *tert-*butyl methyl ether (50 mL) and the solution was stirred at room temperature for 16 h. The bright green precipitate was filtered in air, washed once with a 1 mM solution of DMAP in *tert-*butyl methyl ether (20 mL) and the residue was dried in the vacuum oven at 60 °C for 2 h to give compound **11** (844 mg, 0.968 mmol, 89%). ^1^H NMR (300.1 MHz, C_6_D_6_, 20 °C) δ 19.80 (s, Ru=*CH*), 8.54 (d, ^3^*J*[^1^H^1^H] = 6.4 Hz, 2H), 8.18 (d, ^3^*J*[^1^H^1^H] = 7.0 Hz, 2H), 6.07 (d, ^3^*J*[^1^H^1^H] = 7.0 Hz, 2H), 5.44 (d, ^3^*J*[^1^H^1^H] = 6.4 Hz, 2H, 2 × C_5_N*H*_4_), 8.29 (d, ^3^*J*[^1^H^1^H] = 7.6 Hz, 2H), 7.24 (t, ^3^*J*[^1^H^1^H] = 7.4 Hz, 1H), 7.02 (d, ^3^*J*[^1^H^1^H] = 7.6 Hz, 2H, C_6_*H*_5_), 6.63 (s, 2H), 6.35 (s, 2H, 2 × C_6_*H*_2_), 3.59 (m, 2H), 3.48 (m, 2H, C*H*_2_-C*H*_2_), 3.03 (s, 6H), 2.63 (s, 6H), 2.59 (s, 6H), 2.55 (s, 6H, 4 × N(C*H*_3_)_2_), 2.20 (s, 6H), 1.80 (s, 6H, 2 × C_6_H_2_(C*H*_3_)_2_; ^13^C {^1^H} NMR (75.9 MHz, C_6_D_6_, 20 °C) δ 310.2 (=C*H*), 221.6 (N-*C*-N), 154.1, 153.9, 152.9, 152.5, 150.9, 141.2, 139.0, 127.8, 131.3, 130.9, 129.3, 113.5, 113.0, 107.0, 106.6 (aryl-*C*), 52.3, 51.5 (N-*C*H_2_-*C*H_2_-N), 40.9, 40.7, 38.6 (br), 38.2 (N-*C*H_3_), 22.1 (br), 19.9 (C_6_H_2_(*C*H_3_)_2_); Anal. calcd for C_48_H_72_Cl_2_N_4_PRuS: C, 61.32; H, 7.54; N, 5.96, found: C, 61.40; H, 7.64, N, 5.93.

**Synthesis of (1,3-bis(2’,6’-dimethyl-4’-dimethylaminophenyl)-2-dihydroimidazolidinylidene)bis(4-dimethylaminopyridine)dichloro(phenylthiomethylene)ruthenium(II) (DMAP)_2_Cl_2_(H_2_ITap)Ru=CHSPh (12):** 4-Dimethylaminopyridine (DMAP, 412 mg, 3.38 mmol) was added to a slurry of (PCy_3_)Cl_2_(H_2_ITap)Ru=CHSPh (**9**, 1.237 g, 1.32 mmol) in *tert-*butyl methyl ether (80 mL) and the solution was stirred for 16 h at 50 °C. The grayish-green precipitate was filtered in air, washed once with a 1 mM solution of DMAP in *tert-*butyl methyl ether (20 mL) and the residue was dried in the vacuum oven at 60 °C for 2 h to give compound **12** (1.110 g, 1.23 mmol, 93%).

**NMR specroscopic analysis of (1,3-bis(2’,6’-dimethyl-4’-dimethylaminophenyl)-2-dihydroimidazolidinylidene)bis(4-dimethylaminopyrine)dichloro(phenylthiomethylene)ruthenium(II) (DMAP)_2_Cl_2_(H_2_ITap)Ru=CHSPh (12):** Complex **12** has been found to be low-soluble in a variety of organic solvents including benzene, ether, THF and acetone. Chlorinated solvents such as CH_2_Cl_2_ and CHCl_3_ dramatically improve the complex solubility but have shown to result in significant degradation over a period of several hours. An NMR sample of complex **12** in CDCl_3_ exhibited approx. 10% decomposition over a 24 h period at room temperature as observed by ^1^H NMR spectroscopy. Both, ^1^H NMR and ^13^C NMR spectra, exhibit broadened signals at room temperature due to dynamic processes. ^1^H NMR (400.1 MHz, CDCl_3_, 20 °C) δ 17.33 (s, 1H, Ru=*CH*), 8.22 (br, 2H), 7.73 (br, 2H), 6.56 (br, 2H), 6.49 (br, 2H, 2 × C_5_N*H*_4_), 6.20 (br, 2H), 6.15 (s, 2H, 2 × C_6_*H*_2_), 7.23–7.05 (m, 5H, S-C_6_*H*_5_), 4.10 (m, 2H), 3.96 (m, 2H, C*H*_2_-C*H*_2_), 3.11 (s, 6H), 2.95 (s, 6H), 2.89 (s, 6H), 2.69 (s, 6H, 4 × N(C*H*_3_)_2_), 2.60 (s, 6H), 2.40 (s, 6H, 2 × C_6_H_2_(C*H*_3_)_2_); ^13^C {^1^H} NMR (75.9 MHz, CDCl_3_, 20 °C) δ 287.1 (br, Ru=*C*H), 220.7 (N-*C*-N), 153.8 (br), 153.5 (br), 145.0, 148.9, 148.3 (br), 142.3 (br), 138.4, 128.1, 126.8, 125.9, 112.0, 111.2, 106.2 (2 signals, aryl-*C*), 52.0, 51.2 (br, N-*C*H_2_-*C*H_2_-N), 40.4, 39.8, 38.9 (2 signals, N-*C*H_3_), 20.6 (br), 19.4 (C_6_H_2_(*C*H_3_)_2_). Cooling a solution of complex **12** in CDCl_3_ to −20 °C allowed the observation of two isomers which are in a dynamic equilibrium at room temperature. A detailed analysis of the two isomers is beyond the scope of this manuscript. ^1^H NMR (400.1 MHz, CDCl_3_, −20 °C): δ 17.36, 17.28 (s, Ru=C*H*), 8.48, 8.16, 7.96, 7.62, 6.63, 6.54, 5.96, 5.93 (br, 4 × C_5_N*H*_4_), 6.23, 6.14, 6.04 (4 × C_6_*H*_2_), 7.23–7.05 (S-C_6_*H*_5_), 4.16, 4.01, 3.81 (2 × C*H*_2_-C*H*_2_), 3.15, 2.97, 2.90 (2 signals), 2.84, 2.73, 2.70, 2.59, 2.57, 2.47 (2 signals), 2.39 (8 × N(C*H*_3_)_2_ and 2 × C_6_H_2_(C*H*_3_)_2_); ^13^C {^1^H} NMR (75.9 MHz, CDCl_3_, 20 °C): δ 287.7, 287.4 (Ru=*C*H), 220.0 (N-*C*-N), 155.6, 152.6, 151.9, 150.3, 149.6, 149.5, 148.8, 148.1, 143.4, 141.2, 138.6, 138.2, 137.8, 131.1 129.7, 128.0 (2 signals), 127.3, 126.9, 126.3, 126.0, 125.1, 123.4, 111.7, 111.2, 110.8 106.6, 106.4, 105.5 (2 signals, aryl-*C*), 52.2, 52.0, 51.7, 50.5 (N-*C*H_2_-*C*H_2_-N), 40.6, 40.2, 40.0, 39.7, 39.1, 38.8 (2 signals, N-*C*H_3_), 20.9, 19.8, 19.1 (C_6_H_2_(*C*H_3_)_2_); Anal. calcd for C_44_H_58_Cl_2_N_8_RuS: C, 58.52; H, 6.47; N, 12.41; found: C, 58.26; H, 6.49, N, 11.74.

**Crystal structure determination of complex 12.** Crystals suitable for X-ray diffraction were obtained by layer diffusion of heptane into a THF solution of complex **12** at ambient temperatures over a period of 3 d to yield dark brown prisms. The crystals do not survive away from their solvent for any appreciable period at all, and disintegrate fairly soon after removal from the solvent. A small specimen (0.25 × 0.33 × 0.38 mm) was wedged at the top of a 0.3 mm glass capillary tube while in contact with a small amount of its solvent. The capillary tube was truncated to isolate the sample, sealed with epoxy, and mounted on a pin; the pin was placed on a goniometer head. The crystallographic properties and data were collected using Mo Kα radiation and the charge-coupled area detector (CCD) detector on an Oxford Diffraction Systems Gemini S diffractometer at 300(1) K. A preliminary set of cell constants was calculated from reflections observed on three sets of 5 frames which were oriented approximately in mutually orthogonal directions of reciprocal space. Data collection was carried out using Mo Kα radiation (graphite monochromator) with 8 runs consisting of 511 frames with a frame time of 45.0 s and a crystal-to-CCD distance of 50.000 mm. The runs were collected by omega scans of 1.0 degree width, and at detector position of 28.484, −30.203 degrees in 2θ. The intensity data were corrected for absorption with an analytical correction. Final cell constants were calculated from 5404 stronger reflections from the actual data collection after integration. See Supporting Information File 1 for crystal and refinement information.

**General procedure for ROMP of COE.** Analogous to the procedure described in [35], COE (7.2 μL, 60 μmol) was added via a microliter syringe through a septum to a stock solution of the catalyst (in C_6_D_6_ for **9** and **11**, CDCl_3_ for **12** – 0.5 mM, 0.60 mL, 0.3 μmol) in an NMR tube. The monomer conversion was monitored at 20 °C via ^1^H NMR spectroscopy by integration of the sufficiently separated multiplet signals at δ 5.51 ppm (m, monomer =C*H***-**) and 5.46 ppm (m, polymer, =C*H*-).

**General procedure for RCM of diethyl diallylmalonate (DEDAM).** Analogous to the procedure described in [72], DEDAM (14.6 μL, 60 μmol) was added via microliter syringe through a septum to a stock solution of the catalyst (in C_6_D_6_ for **9** and **11**, CDCl_3_ for **12** – 1.0 mM, 0.60 mL, 0.6 μmol) in a NMR tube. The substrate conversion was monitored at 20 °C via ^1^H NMR spectroscopy by integration of the sufficiently separated multiplet signals at δ 2.78 ppm (m, allyl-C*H*_2_, DEDAM) and 3.13 ppm (m, ring-C*H*_2_, cyclopentene derivative).

**General procedure for the RCM of diallylmalonic acid (DAMA).** Analogous to the procedure described in [72], the catalyst (8 μmol) and DAM (36.8 mg, 0.20 mmol) were dissolved in the 0.1 M HCl_aq_ (2.0 mL) under inert gas conditions and the solution was heated to 50 °C under stirring. An aliquot (0.3 mL) was taken after 30 min and 60 min, quenched with ethyl vinyl ether, dried under vacuum, and the monomer conversion was monitored via ^1^H NMR spectroscopy (300.1 MHz, 20 °C, D_2_O) by integration of the signals δ 2.58 (DAMA-CH_2_) and δ 2.98 ppm (cyclopentene-C*H*_2_). The aliquots taken after 60 min indicated the same conversion level as those taken after 30 min.

**General procedure for the preparation of the polymer dispersions using DCPD or DCPD/COE mixtures with complexes 11 and 12.** A mixture of 73.1 g of water, 8.3 g of a 10% (by strength) solution of PEG-30 cetyl stearyl ether (Emulgin^®^ B3) as charge-neutral surfactant, 0.75 g of *n-*hexadecane and 15.3 g (116 mmol) DCPD or 8.40 g (63.5 mmol) DCPD + 7.2 g COE (65.3 mmol) was stirred vigorously for 1 h under a nitrogen atmosphere before it was further homogenized using an ultrasonic probe for 5 min. Then a solution of catalyst (20.1 mg (**11**) or 20.6 mg (**12**), 0.023 mmol) in 13.6 g of 0.1 M aqueous HCl was added dropwise to the resulting microemulsion under constant stirring over a period of 1 min. The reaction mixture was stirred then at the reaction temperature (35 °C, 55 °C, 65 °C) for 2 h. After that time, the emulsion was pressed through a 20 μm pore filter and an aliquot of approx. 0.8 g was taken from the emulsion for solid residue analysis.

**Crystallographic data:** Crystallographic data for structure **12** has been deposited with the Cambridge Crystallographic Data Centre (CCDC 1404596). Copies of the data can be obtained, free of charge, on application to the Director, CCDC, 12 Union Road, Cambridge CB2 1EZ, United Kingdom (Fax: 44-1223-336033 or e-mail: deposit@ccdc.cam.ac.uk).

## Supporting Information

File 1 Crystallographic data of compound **12**.

File 2 ^1^H, ^13^C and ^31^P NMR spectra of the synthesized Ru-complexes **9**, **11** and **12** as well as kinetic experimental data.
