# Supplementary material for: Hexacoordinate Ru-based olefin metathesis catalysts with pH-responsive N-heterocyclic carbene (NHC) and N-donor ligands for ROMP reactions in non-aqueous, aqueous and emulsion conditions
Source: Beilstein J Org Chem. 2015 Oct 21;11:1960–72. doi: 10.3762/bjoc.11.212 (PMC4660970; doi:10.3762/bjoc.11.212)
Supplement: File 1 — Crystallographic data of compound 12. [file Beilstein_J_Org_Chem-11-1960-s001.pdf]

Supporting Information

for

Hexacoordinate Ru-based olefin metathesis catalysts with pH-responsive N-heterocyclic carbene (NHC) and N-donor ligands for ROMP reactions in non-aqueous, aqueous and emulsion conditions

Shawna L. Balof [1], K. Owen Nix[2], Matthew S. Olliff [2], Sarah E. Roessler [2], Arpita Saha[2], Kevin B. Müller [3], Ulrich Behrens [4], Edward J. Valente[5] and Hans-Jörg Schanz[2]*

Address: [1]Department of Chemistry & Biochemistry, The University of Southern Mississippi, 118 College Drive, Hattiesburg, MS 39406-5043, USA, [2]Department of Chemistry, Georgia Southern University, 521 College of Education Drive, Statesboro, GA 30458-8064, USA, [3]BASF SE, G-PM/PD - F206, 67056 Ludwigshafen, Germany, [4]BASF SE, Basic Chemicals Research, GCB/C – M313, 67056 Ludwigshafen, Germany and [5]Department of Chemistry, University of Portland, 5000 N. Willamette Blvd., Portland, OR 97203, USA

*Corresponding author
Email: Hans-Jörg Schanz - hschanz@georgiasouthern.edu

Crystallographic data of compound 12

Internal Reference Identifier: Complex **12** (2015 Revision)

CRYSTAL STRUCTURE REPORT

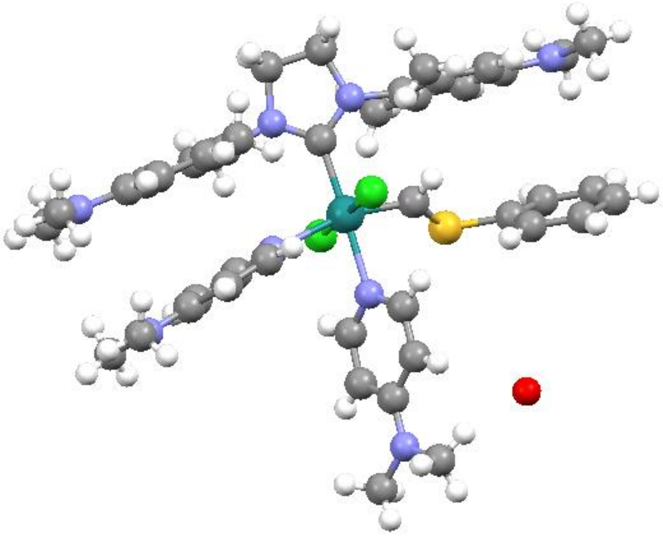

March 22, 2010; revised June 2, 2015

Prepared for Dr. Hans-Jorg Schanz

Georgia Southern University

formerly University of Southern Mississippi

Edward J. Valente, Ph. D.

University of Portland Diffraction Facility

Department of Chemistry & Biochemistry

University of Portland

MSC 28

5000 North Willamette Boulevard

Portland, OR 97203

Thermal ellipsoid plot.

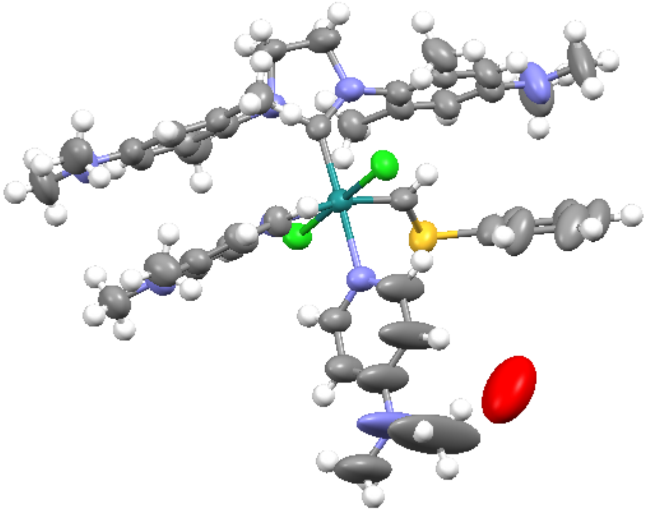

Uniform thermal ellipsoids.

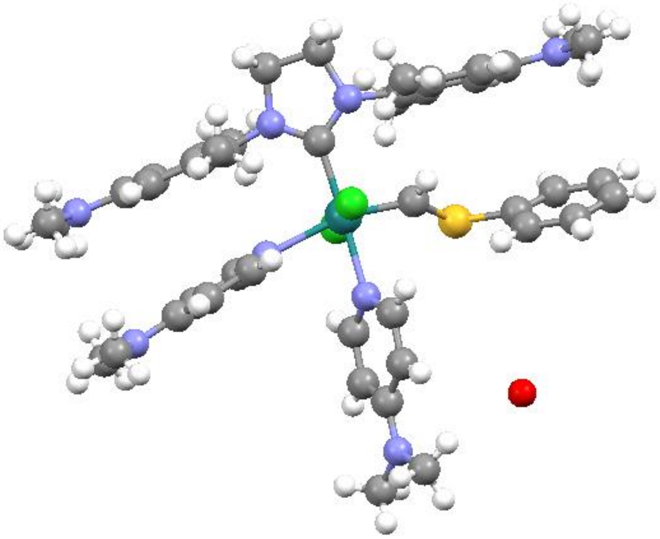

Data collection

Dark red or brown prisms were provided in an 5 mm nmr tube in contact with their liquor (reported as thf and heptane) The crystals do not survive away from their solvent for any appreciable period at all, and disintegrate fairly soon after removal from the solvent. A small specimen (0.25 x 0.33 x 0.38 mm) was wedged at the top of an 0.3 mm glass capillary tube while in contact with a small amount of its solvent. The capillary tube was truncated to isolate the sample, sealed with epoxy, and mounted on a pin; the pin was placed on a goniometer head. The crystallographic properties and data were collected using MoKα radiation and the charge-coupled area detector (CCD) detector on an Oxford Diffraction Systems Gemini S diffractometer at 300(1)K (1). A preliminary set of cell constants was calculated from reflections observed on three sets of 5 frames which were oriented approximately in mutually orthogonal directions of reciprocal space. Data collection was carried out using MoKα radiation (graphite monochromator) with 8 runs consisting of 511 frames with a frame time of 45.0 sec and a crystal-to-CCD distance of 50.000mm. The runs were collected by omega scans of 1.0 degree width, and at detector position of 28.484, -30.203 degrees in 2θ. The intensity data were corrected for absorption with an analytical correction (4). Final cell constants were calculated from 5404 stronger reflections from the actual data collection after integration. See Table 1 for crystal and refinement information.

Structure solution and refinement

The crystal is triclinic, space group P-1 (#2) as determined from the cell geometry, lack of systematic absences, reflections statistics, and successful solution and refinement. The structure was solved using by direct methods in SHELXS-86(2b), and all non-H atoms were found in the E-map. Refinements were done using SHELXL-97(2a). All non-H atoms were refined and with anisotropic librational factors. H-atoms were observable in difference electron density maps, and placed in idealize positions; all were refined as riding atoms with relative isotropic displacement parameters of 120% of the U(eq) of the attached atom. The coordinated p-dimethyaminopyridine is quite thermal perpendicular to the mean ligand plane, and there is a low density site (modeled as a water oxygen) in its vicinity. It is perhaps the case that the solvent has largely departed from the crystal, but a fairly thermal water has been retained in the site, which is near a cell center of symmetry, suggesting that pairs are H-bonded. The final full-matrix least-squares refinement converged to $R1 = 0.0632$ (6435 reflections, F^2, $I > 2\sigma(I)$); R1 = 0.1112 and wR2 = 0.1763 for all 10376 data (to 2θ 55.0°), 514 parameters, 0 restraints, goodness-of-fit (S) = 1.012 , and no extinction.

Structure description

The asymmetric unit consists of the ruthenium (III) cation in approximately octahedral coordination geometry: two chlorides, two 4-dimethylaminopyridines, a pyrazole carbine ligand and a thiocarbene ligand. There are no remarkable intermolecular contacts. Solvate waters are probably H-bonded across centers of symmetry, but the site is very thermal or partially occupied. The axial dimethylamino pyridine is particularly thermal.

Other Information

Data collection and structure solution were conducted at the University of Portland Diffraction Facility, 112A Swindells Hall, Department of Chemistry, University of Portland, 5000 N. Willamette Blvd., Portland, OR, 97203. All calculations were performed using Pentium computers using the current SHELX suite of programs. All publications arising from this report by participating senior personnel in the acquisition grant must include Edward J. Valente as a co-author, and "gratefully acknowledge the support of NSF grant MRI 0618148.

Relevant References

1. CrysalisPro (2007 and 2014), Version 171.32.5, Oxford Diffraction Ltd., Abingdon, Oxfordshire, OX14 4RX, United Kingdom.

2a. SHELX97 [Includes SHELXS97, SHELXL97, CIFTAB] - Programs for Crystal Structure Analysis (Release 97-2). G. M. Sheldrick, Institüt für Anorganische Chemie der Universität, Tammanstrasse 4, D-3400 Göttingen, Germany, 1998.

2b. SHELXS86 - G. M. Sheldrick, In "Crystallographic Computing 3", Ed. G. M. Sheldrick, C. Kruger and R. Goddard, Oxford University Press. pp. 175-189, 1985.

SHELXS86 - Program for Crystal Structure solution. G. M. Sheldrick, Institüt für Anorganische Chemie der Universität, Tammanstrasse 4, D-3400 Göttingen, Germany, 1986

3. MERCURY (version 3.5.1) Cambridge Crystallographic Data Center. (shareware).

ORTEP3 for Windows - L. J. Farrugia, J. Appl. Crystallogr. 1997, 30, 565.

4. ABSORPTION CORRECTION (ANALYTICAL) - C. Katayama, Acta Crystallogr., Sect A 1986, 42, 19-23.

5. Wingx L.J. Farrugia, J. Appl. Cryst., 1999, 32, 837-838.

6. PLATON/PLUTON - (a) A. L. Spek, Acta Crystallogr., Sect A 1990, 46, C34. (b) PLATON, A Multipurpose Crystallographic Tool, Utrecht University, Utrecht, The Netherlands, A. L. Spek, 1998.

Relevant Equations used in this report:

$$R_{int} = \Sigma\ |F_o^2 - \langle F_o^2 \rangle| / \Sigma|F_o^2|$$

$$R_1 = \Sigma||F_o|-|F_c|| / \Sigma|F_o|$$

$$wR2 = [\Sigma[w(F_o^2-F_c^2)^2] / \Sigma\ [w(F_o^2)^2]]^{1/2}$$

$$\text{where } w = q / [\sigma^2 (F_o^2) + (a*P)^2 + b*P + d + e*\sin(\theta)]$$

$$\text{GooF} = S = [\Sigma[w(F_o^2-F_c^2)^2] / (n-p)]^{1/2}$$

Table 1. Crystal data and structure refinement for **12**.

Empirical formula	C44 H60 Cl2 N8 O S Ru
Formula weight	921.03
Temperature	300(2) K
Wavelength	0.71073 A
Crystal system, space group	Triclinic, P -1
Unit cell dimensions	a = 10.2337(6) A alpha = 86.256(4) deg.
	b = 12.3509(6) A beta = 88.726(4) deg.
	c = 18.2931(9) A gamma = 78.700(4) deg.
Volume	2262.4(2) A^3
Z, Calculated density	2, 1.349 Mg/m^3
Absorption coefficient	0.553 mm^-1
F(000)	960
Crystal size	0.25 x 0.33 x 0.38 mm
Theta range for data collection	3.34 to 27.50 deg.
Limiting indices	-13<=h<=12, -15<=k<=16, -23<=l<=23
Reflections collected / unique	21143 / 10376 [R(int) = 0.0540]
Completeness to theta = 27.50	99.8 %
Absorption correction	Semi-empirical from equivalents
Max. and min. transmission	1 and 0.77324
Refinement method	Full-matrix least-squares on F^2
Data / restraints / parameters	10376 / 0 / 514
Goodness-of-fit on F^2	1.012
Final R indices [I>2sigma(I)]	R1 = 0.0632, wR2 = 0.1546
R indices (all data)	R1 = 0.1112, wR2 = 0.1763
Largest diff. peak and hole	0.696 and -0.544 e.A^-3

Table 2. Atomic coordinates (x 10^4) and equivalent isotropic displacement parameters (A^2 x 10^3) for **12.** U(eq) is defined as one third of the trace of the orthogonalized Uij tensor.

Atom	x	y	z	U(eq)
C(1)	9074(5)	6389(4)	2078(2)	57(1)
C(2)	11407(5)	6177(5)	1272(3)	69(1)
C(3)	12728(7)	5805(5)	1354(3)	87(2)
C(4)	13479(7)	5231(6)	807(4)	103(2)
C(5)	12886(9)	5048(6)	175(4)	111(2)
C(6)	11577(9)	5456(7)	91(4)	120(3)
C(7)	10806(7)	6026(6)	639(3)	102(2)
C(8)	4361(5)	8091(4)	2402(2)	60(1)
C(9)	3148(6)	8783(4)	2477(3)	69(1)
C(10)	2993(5)	9605(4)	2973(3)	62(1)
C(11)	4144(5)	9653(4)	3362(3)	62(1)
C(12)	5282(5)	8921(4)	3260(2)	57(1)
C(13)	628(6)	10175(5)	2704(4)	89(2)
C(14)	1696(7)	11144(5)	3592(3)	90(2)
C(15)	5627(5)	6545(4)	4174(2)	54(1)
C(16)	5835(5)	7184(4)	4748(2)	61(1)
C(17)	4746(6)	7874(4)	5038(3)	70(1)
C(18)	3467(6)	7931(4)	4795(3)	71(1)
C(19)	3273(5)	7274(4)	4228(3)	63(1)
C(20)	4345(5)	6567(4)	3936(2)	55(1)
C(21)	7207(6)	7137(5)	5037(3)	85(2)
C(22)	2550(9)	9326(6)	5659(4)	126(3)
C(23)	1039(7)	8594(6)	4855(4)	107(2)
C(24)	4102(5)	5817(4)	3357(3)	70(1)
C(25)	9431(5)	4305(4)	2914(2)	56(1)
C(26)	10620(5)	4504(4)	3177(2)	56(1)
C(27)	11801(6)	4022(4)	2857(3)	70(1)
C(28)	11850(6)	3322(4)	2287(3)	73(1)
C(29)	10645(6)	3139(4)	2041(3)	75(2)
C(30)	9433(5)	3600(4)	2351(3)	64(1)
C(31)	10624(5)	5257(4)	3797(3)	69(1)
C(32)	14311(7)	2913(7)	2326(5)	129(3)
C(33)	13119(8)	2072(7)	1414(5)	143(3)
C(34)	8170(6)	3286(4)	2105(3)	87(2)
C(35)	7479(4)	5734(4)	3318(2)	49(1)
C(36)	7248(11)	8276(6)	1014(3)	158(4)
C(37)	7036(19)	9138(7)	495(4)	304(11)
C(38)	6984(13)	10226(7)	660(4)	181(5)
C(39)	7210(8)	10327(5)	1385(3)	110(3)
C(40)	7385(6)	9425(4)	1868(3)	74(2)
C(41)	6713(14)	12192(7)	330(5)	217(6)
C(42)	6620(30)	10938(11)	-590(6)	560(30)
C(84)	6867(6)	4647(4)	4335(3)	78(2)
C(85)	7936(6)	3942(4)	3920(3)	83(2)
N(1)	5457(4)	8095(3)	2799(2)	56(1)
N(2)	1802(5)	10302(4)	3063(2)	79(1)
N(3)	2361(6)	8619(4)	5099(3)	100(2)
N(4)	6685(4)	5725(3)	3915(2)	56(1)

N(5)	8212(4)	4697(3)	3302(2)	58(1)
N(6)	13069(6)	2814(5)	1998(3)	109(2)
N(7)	7365(4)	8405(3)	1713(2)	63(1)
N(8)	6818(18)	11080(7)	161(4)	357(10)
S(1)	10491(1)	6915(1)	1989(1)	73(1)
Cl(1)	8597(1)	8004(1)	3349(1)	59(1)
Cl(2)	6386(1)	6018(1)	1730(1)	61(1)
Ru	7482(1)	7018(1)	2544(1)	47(1)
O(1W)	9750(9)	9284(9)	-491(4)	262(5)

Table 3. Bond lengths [A] and angles [deg] for **12**.

C(1)-S(1)	1.701(5)
C(1)-Ru	1.874(5)
C(1)-H(1)	0.9300
C(2)-C(3)	1.348(8)
C(2)-C(7)	1.363(7)
C(2)-S(1)	1.789(5)
C(3)-C(4)	1.393(8)
C(3)-H(3)	0.9300
C(4)-C(5)	1.368(10)
C(4)-H(4)	0.9300
C(5)-C(6)	1.344(10)
C(5)-H(5)	0.9300
C(6)-C(7)	1.402(9)
C(6)-H(6)	0.9300
C(7)-H(7)	0.9300
C(8)-N(1)	1.350(5)
C(8)-C(9)	1.372(7)
C(8)-H(8)	0.9300
C(9)-C(10)	1.388(7)
C(9)-H(9)	0.9300
C(10)-N(2)	1.362(6)
C(10)-C(11)	1.403(7)
C(11)-C(12)	1.345(6)
C(11)-H(11)	0.9300
C(12)-N(1)	1.348(5)
C(12)-H(12)	0.9300
C(13)-N(2)	1.422(7)
C(13)-H(13A)	0.9600
C(13)-H(13B)	0.9600
C(13)-H(13C)	0.9600
C(14)-N(2)	1.451(7)
C(14)-H(14A)	0.9600
C(14)-H(14B)	0.9600
C(14)-H(14C)	0.9600
C(15)-C(20)	1.386(6)
C(15)-C(16)	1.399(6)
C(15)-N(4)	1.426(6)
C(16)-C(17)	1.382(7)
C(16)-C(21)	1.501(7)
C(17)-C(18)	1.379(8)
C(17)-H(17)	0.9300
C(18)-C(19)	1.398(7)
C(18)-N(3)	1.403(7)

C(19)-C(20)	1.383(6)
C(19)-H(19)	0.9300
C(20)-C(24)	1.507(6)
C(21)-H(21A)	0.9600
C(21)-H(21B)	0.9600
C(21)-H(21C)	0.9600
C(22)-N(3)	1.429(8)
C(22)-H(22A)	0.9600
C(22)-H(22B)	0.9600
C(22)-H(22C)	0.9600
C(23)-N(3)	1.441(9)
C(23)-H(23A)	0.9600
C(23)-H(23B)	0.9600
C(23)-H(23C)	0.9600
C(24)-H(24A)	0.9600
C(24)-H(24B)	0.9600
C(24)-H(24C)	0.9600
C(25)-C(30)	1.389(6)
C(25)-C(26)	1.390(7)
C(25)-N(5)	1.437(6)
C(26)-C(27)	1.375(6)
C(26)-C(31)	1.514(6)
C(27)-C(28)	1.391(7)
C(27)-H(27)	0.9300
C(28)-C(29)	1.387(7)
C(28)-N(6)	1.391(7)
C(29)-C(30)	1.386(7)
C(29)-H(29)	0.9300
C(30)-C(34)	1.506(7)
C(31)-H(31A)	0.9600
C(31)-H(31B)	0.9600
C(31)-H(31C)	0.9600
C(32)-N(6)	1.448(9)
C(32)-H(32A)	0.9600
C(32)-H(32B)	0.9600
C(32)-H(32C)	0.9600
C(33)-N(6)	1.446(8)
C(33)-H(33A)	0.9600
C(33)-H(33B)	0.9600
C(33)-H(33C)	0.9600
C(34)-H(34A)	0.9600
C(34)-H(34B)	0.9600
C(34)-H(34C)	0.9600
C(35)-N(4)	1.347(5)
C(35)-N(5)	1.353(5)
C(35)-Ru	2.057(4)
C(36)-N(7)	1.309(7)
C(36)-C(37)	1.367(9)
C(36)-H(36)	0.9300
C(37)-C(38)	1.388(10)
C(37)-H(37)	0.9300
C(38)-N(8)	1.336(9)
C(38)-C(39)	1.368(9)
C(39)-C(40)	1.362(7)
C(39)-H(39)	0.9300
C(40)-N(7)	1.314(6)
C(40)-H(40)	0.9300
C(41)-N(8)	1.410(10)

C(41)-H(41A)	0.9600
C(41)-H(41B)	0.9600
C(41)-H(41C)	0.9600
C(42)-N(8)	1.420(12)
C(42)-H(42A)	0.9600
C(42)-H(42B)	0.9600
C(42)-H(42C)	0.9600
C(84)-N(4)	1.475(5)
C(84)-C(85)	1.486(7)
C(84)-H(84A)	0.9700
C(84)-H(84B)	0.9700
C(85)-N(5)	1.475(6)
C(85)-H(85A)	0.9700
C(85)-H(85B)	0.9700
N(1)-Ru	2.289(4)
N(7)-Ru	2.201(4)
Cl(1)-Ru	2.4091(11)
Cl(2)-Ru	2.4202(11)

S(1)-C(1)-Ru	127.1(3)
S(1)-C(1)-H(1)	116.5
Ru-C(1)-H(1)	116.5
C(3)-C(2)-C(7)	120.0(5)
C(3)-C(2)-S(1)	118.7(4)
C(7)-C(2)-S(1)	121.3(5)
C(2)-C(3)-C(4)	120.5(6)
C(2)-C(3)-H(3)	119.8
C(4)-C(3)-H(3)	119.8
C(5)-C(4)-C(3)	120.3(7)
C(5)-C(4)-H(4)	119.8
C(3)-C(4)-H(4)	119.8
C(6)-C(5)-C(4)	118.5(6)
C(6)-C(5)-H(5)	120.7
C(4)-C(5)-H(5)	120.7
C(5)-C(6)-C(7)	121.8(6)
C(5)-C(6)-H(6)	119.1
C(7)-C(6)-H(6)	119.1
C(2)-C(7)-C(6)	118.8(7)
C(2)-C(7)-H(7)	120.6
C(6)-C(7)-H(7)	120.6
N(1)-C(8)-C(9)	125.6(4)
N(1)-C(8)-H(8)	117.2
C(9)-C(8)-H(8)	117.2
C(8)-C(9)-C(10)	119.9(5)
C(8)-C(9)-H(9)	120.1
C(10)-C(9)-H(9)	120.1
N(2)-C(10)-C(9)	121.4(5)
N(2)-C(10)-C(11)	123.5(5)
C(9)-C(10)-C(11)	115.1(5)
C(12)-C(11)-C(10)	120.5(4)
C(12)-C(11)-H(11)	119.7
C(10)-C(11)-H(11)	119.7
C(11)-C(12)-N(1)	125.9(5)
C(11)-C(12)-H(12)	117.0
N(1)-C(12)-H(12)	117.0
N(2)-C(13)-H(13A)	109.5
N(2)-C(13)-H(13B)	109.5
H(13A)-C(13)-H(13B)	109.5

N(2)-C(13)-H(13C)	109.5
H(13A)-C(13)-H(13C)	109.5
H(13B)-C(13)-H(13C)	109.5
N(2)-C(14)-H(14A)	109.5
N(2)-C(14)-H(14B)	109.5
H(14A)-C(14)-H(14B)	109.5
N(2)-C(14)-H(14C)	109.5
H(14A)-C(14)-H(14C)	109.5
H(14B)-C(14)-H(14C)	109.5
C(20)-C(15)-C(16)	120.0(4)
C(20)-C(15)-N(4)	118.5(4)
C(16)-C(15)-N(4)	120.5(4)
C(17)-C(16)-C(15)	118.3(5)
C(17)-C(16)-C(21)	120.6(5)
C(15)-C(16)-C(21)	121.0(5)
C(18)-C(17)-C(16)	122.5(5)
C(18)-C(17)-H(17)	118.8
C(16)-C(17)-H(17)	118.8
C(17)-C(18)-C(19)	118.4(5)
C(17)-C(18)-N(3)	122.3(5)
C(19)-C(18)-N(3)	119.3(6)
C(20)-C(19)-C(18)	120.2(5)
C(20)-C(19)-H(19)	119.9
C(18)-C(19)-H(19)	119.9
C(19)-C(20)-C(15)	120.4(4)
C(19)-C(20)-C(24)	119.2(5)
C(15)-C(20)-C(24)	120.4(4)
C(16)-C(21)-H(21A)	109.5
C(16)-C(21)-H(21B)	109.5
H(21A)-C(21)-H(21B)	109.5
C(16)-C(21)-H(21C)	109.5
H(21A)-C(21)-H(21C)	109.5
H(21B)-C(21)-H(21C)	109.5
N(3)-C(22)-H(22A)	109.5
N(3)-C(22)-H(22B)	109.5
H(22A)-C(22)-H(22B)	109.5
N(3)-C(22)-H(22C)	109.5
H(22A)-C(22)-H(22C)	109.5
H(22B)-C(22)-H(22C)	109.5
N(3)-C(23)-H(23A)	109.5
N(3)-C(23)-H(23B)	109.5
H(23A)-C(23)-H(23B)	109.5
N(3)-C(23)-H(23C)	109.5
H(23A)-C(23)-H(23C)	109.5
H(23B)-C(23)-H(23C)	109.5
C(20)-C(24)-H(24A)	109.5
C(20)-C(24)-H(24B)	109.5
H(24A)-C(24)-H(24B)	109.5
C(20)-C(24)-H(24C)	109.5
H(24A)-C(24)-H(24C)	109.5
H(24B)-C(24)-H(24C)	109.5
C(30)-C(25)-C(26)	120.7(5)
C(30)-C(25)-N(5)	119.6(5)
C(26)-C(25)-N(5)	119.0(4)
C(27)-C(26)-C(25)	119.1(4)
C(27)-C(26)-C(31)	120.0(5)
C(25)-C(26)-C(31)	120.9(4)
C(26)-C(27)-C(28)	122.2(5)

C(26)-C(27)-H(27)	118.9
C(28)-C(27)-H(27)	118.9
C(29)-C(28)-C(27)	117.1(5)
C(29)-C(28)-N(6)	122.3(5)
C(27)-C(28)-N(6)	120.5(6)
C(30)-C(29)-C(28)	122.5(5)
C(30)-C(29)-H(29)	118.8
C(28)-C(29)-H(29)	118.8
C(29)-C(30)-C(25)	118.3(5)
C(29)-C(30)-C(34)	119.9(5)
C(25)-C(30)-C(34)	121.7(5)
C(26)-C(31)-H(31A)	109.5
C(26)-C(31)-H(31B)	109.5
H(31A)-C(31)-H(31B)	109.5
C(26)-C(31)-H(31C)	109.5
H(31A)-C(31)-H(31C)	109.5
H(31B)-C(31)-H(31C)	109.5
N(6)-C(32)-H(32A)	109.5
N(6)-C(32)-H(32B)	109.5
H(32A)-C(32)-H(32B)	109.5
N(6)-C(32)-H(32C)	109.5
H(32A)-C(32)-H(32C)	109.5
H(32B)-C(32)-H(32C)	109.5
N(6)-C(33)-H(33A)	109.5
N(6)-C(33)-H(33B)	109.5
H(33A)-C(33)-H(33B)	109.5
N(6)-C(33)-H(33C)	109.5
H(33A)-C(33)-H(33C)	109.5
H(33B)-C(33)-H(33C)	109.5
C(30)-C(34)-H(34A)	109.5
C(30)-C(34)-H(34B)	109.5
H(34A)-C(34)-H(34B)	109.5
C(30)-C(34)-H(34C)	109.5
H(34A)-C(34)-H(34C)	109.5
H(34B)-C(34)-H(34C)	109.5
N(4)-C(35)-N(5)	105.8(4)
N(4)-C(35)-Ru	127.3(3)
N(5)-C(35)-Ru	126.7(3)
N(7)-C(36)-C(37)	123.3(6)
N(7)-C(36)-H(36)	118.3
C(37)-C(36)-H(36)	118.3
C(36)-C(37)-C(38)	122.7(6)
C(36)-C(37)-H(37)	118.7
C(38)-C(37)-H(37)	118.7
N(8)-C(38)-C(39)	123.2(7)
N(8)-C(38)-C(37)	123.9(7)
C(39)-C(38)-C(37)	112.8(6)
C(40)-C(39)-C(38)	120.5(6)
C(40)-C(39)-H(39)	119.7
C(38)-C(39)-H(39)	119.7
N(7)-C(40)-C(39)	126.2(5)
N(7)-C(40)-H(40)	116.9
C(39)-C(40)-H(40)	116.9
N(8)-C(41)-H(41A)	109.5
N(8)-C(41)-H(41B)	109.5
H(41A)-C(41)-H(41B)	109.5
N(8)-C(41)-H(41C)	109.5
H(41A)-C(41)-H(41C)	109.5

H(41B)-C(41)-H(41C)	109.5
N(8)-C(42)-H(42A)	109.5
N(8)-C(42)-H(42B)	109.5
H(42A)-C(42)-H(42B)	109.5
N(8)-C(42)-H(42C)	109.5
H(42A)-C(42)-H(42C)	109.5
H(42B)-C(42)-H(42C)	109.5
N(4)-C(84)-C(85)	103.1(4)
N(4)-C(84)-H(84A)	111.1
C(85)-C(84)-H(84A)	111.1
N(4)-C(84)-H(84B)	111.1
C(85)-C(84)-H(84B)	111.1
H(84A)-C(84)-H(84B)	109.1
N(5)-C(85)-C(84)	103.0(4)
N(5)-C(85)-H(85A)	111.2
C(84)-C(85)-H(85A)	111.2
N(5)-C(85)-H(85B)	111.2
C(84)-C(85)-H(85B)	111.2
H(85A)-C(85)-H(85B)	109.1
C(12)-N(1)-C(8)	112.9(4)
C(12)-N(1)-Ru	123.9(3)
C(8)-N(1)-Ru	122.2(3)
C(10)-N(2)-C(13)	122.1(5)
C(10)-N(2)-C(14)	119.1(5)
C(13)-N(2)-C(14)	118.5(5)
C(18)-N(3)-C(22)	119.8(6)
C(18)-N(3)-C(23)	119.8(5)
C(22)-N(3)-C(23)	120.3(6)
C(35)-N(4)-C(15)	131.2(4)
C(35)-N(4)-C(84)	114.1(4)
C(15)-N(4)-C(84)	114.5(3)
C(35)-N(5)-C(25)	129.6(4)
C(35)-N(5)-C(85)	113.9(4)
C(25)-N(5)-C(85)	114.0(4)
C(28)-N(6)-C(33)	120.5(6)
C(28)-N(6)-C(32)	121.0(5)
C(33)-N(6)-C(32)	118.2(6)
C(36)-N(7)-C(40)	114.2(4)
C(36)-N(7)-Ru	122.1(4)
C(40)-N(7)-Ru	123.8(3)
C(38)-N(8)-C(41)	124.1(8)
C(38)-N(8)-C(42)	121.6(9)
C(41)-N(8)-C(42)	114.1(8)
C(1)-S(1)-C(2)	104.3(2)
C(1)-Ru-C(35)	96.22(17)
C(1)-Ru-N(7)	86.32(17)
C(35)-Ru-N(7)	176.86(16)
C(1)-Ru-N(1)	163.28(15)
C(35)-Ru-N(1)	99.66(15)
N(7)-Ru-N(1)	77.65(14)
C(1)-Ru-Cl(1)	93.02(14)
C(35)-Ru-Cl(1)	92.42(12)
N(7)-Ru-Cl(1)	89.29(11)
N(1)-Ru-Cl(1)	91.53(10)
C(1)-Ru-Cl(2)	86.33(14)
C(35)-Ru-Cl(2)	87.58(12)
N(7)-Ru-Cl(2)	90.74(11)
N(1)-Ru-Cl(2)	89.12(10)

Cl(1)-Ru-Cl(2)	179.35(4)

Table 4. Anisotropic displacement parameters (A^2 x 10^3) for **12**.
The anisotropic displacement factor exponent takes the form:
-2 pi^2 [h^2 a*^2 U11 + ... + 2 h k a* b* U12]

Atom	U11	U22	U33	U23	U13	U12
C(1)	75(3)	49(3)	53(2)	-1(2)	-2(2)	-26(2)
C(2)	64(3)	85(4)	67(3)	-11(3)	11(2)	-36(3)
C(3)	92(5)	114(5)	65(3)	-11(3)	6(3)	-44(4)
C(4)	96(5)	124(6)	96(5)	-27(4)	31(4)	-37(4)
C(5)	122(6)	143(6)	89(5)	-50(4)	43(4)	-70(5)
C(6)	117(6)	190(8)	85(4)	-51(5)	15(4)	-96(6)
C(7)	84(4)	174(7)	66(4)	-36(4)	10(3)	-58(5)
C(8)	64(3)	65(3)	51(3)	-12(2)	-12(2)	-11(2)
C(9)	74(4)	64(3)	70(3)	-5(2)	-24(3)	-15(3)
C(10)	70(3)	52(3)	64(3)	1(2)	-2(2)	-11(2)
C(11)	70(3)	52(3)	66(3)	-15(2)	0(2)	-18(2)
C(12)	66(3)	51(3)	57(3)	-6(2)	-5(2)	-19(2)
C(13)	69(4)	77(4)	122(5)	-7(3)	-4(3)	-10(3)
C(14)	93(5)	74(4)	97(4)	-12(3)	9(3)	-2(3)
C(15)	64(3)	55(3)	45(2)	0(2)	7(2)	-18(2)
C(16)	78(3)	68(3)	44(2)	-7(2)	-3(2)	-29(3)
C(17)	101(5)	59(3)	53(3)	-13(2)	6(3)	-23(3)
C(18)	88(4)	58(3)	63(3)	-2(2)	22(3)	-10(3)
C(19)	68(3)	58(3)	62(3)	2(2)	8(2)	-16(2)
C(20)	64(3)	55(3)	49(2)	-1(2)	7(2)	-20(2)
C(21)	104(5)	95(4)	62(3)	-5(3)	-20(3)	-35(4)
C(22)	168(8)	93(5)	106(5)	-38(4)	31(5)	9(5)
C(23)	100(5)	93(5)	110(5)	10(4)	27(4)	20(4)
C(24)	69(4)	80(4)	71(3)	-18(3)	5(2)	-32(3)
C(25)	61(3)	47(3)	60(3)	-2(2)	2(2)	-14(2)
C(26)	60(3)	47(3)	63(3)	-5(2)	-5(2)	-10(2)
C(27)	67(3)	61(3)	85(3)	-17(3)	9(3)	-19(3)
C(28)	73(4)	61(3)	88(4)	-19(3)	16(3)	-21(3)
C(29)	90(4)	58(3)	82(4)	-24(3)	6(3)	-21(3)
C(30)	72(4)	48(3)	75(3)	-9(2)	-4(3)	-18(2)
C(31)	73(4)	72(3)	65(3)	-16(2)	-7(2)	-14(3)
C(32)	61(4)	152(7)	179(8)	-65(6)	24(5)	-14(4)
C(33)	121(7)	147(7)	173(7)	-105(6)	62(5)	-34(5)
C(34)	84(4)	67(4)	112(4)	-27(3)	-16(3)	-14(3)
C(35)	54(3)	54(3)	45(2)	-7(2)	0(2)	-23(2)
C(36)	359(14)	87(5)	52(4)	6(3)	-36(5)	-96(7)
C(37)	800(30)	104(6)	49(4)	17(4)	-66(9)	-186(12)
C(38)	406(16)	98(6)	59(4)	22(4)	-42(6)	-101(8)
C(39)	204(8)	70(4)	66(4)	-3(3)	-5(4)	-54(4)
C(40)	113(5)	61(3)	54(3)	2(2)	-7(3)	-32(3)
C(41)	420(20)	94(7)	139(8)	45(6)	-15(10)	-75(9)
C(42)	1470(80)	182(12)	88(8)	63(7)	-200(20)	-290(30)
C(84)	92(4)	59(3)	78(3)	17(3)	14(3)	-15(3)
C(85)	83(4)	62(3)	99(4)	19(3)	17(3)	-10(3)
N(1)	71(3)	56(2)	47(2)	-8(2)	1(2)	-22(2)
N(2)	76(3)	72(3)	89(3)	-19(2)	-3(2)	-10(3)
N(3)	105(5)	91(4)	99(4)	-31(3)	30(3)	-5(3)
N(4)	69(3)	49(2)	51(2)	4(2)	6(2)	-14(2)

N(5)	61(2)	51(2)	64(2)	4(2)	4(2)	-15(2)
N(6)	86(4)	101(4)	147(5)	-62(4)	36(4)	-22(3)
N(7)	93(3)	55(2)	47(2)	-1(2)	-2(2)	-27(2)
N(8)	920(30)	109(6)	80(5)	43(4)	-88(10)	-198(12)
S(1)	74(1)	88(1)	67(1)	-20(1)	10(1)	-40(1)
Cl(1)	69(1)	57(1)	55(1)	-8(1)	-9(1)	-24(1)
Cl(2)	68(1)	64(1)	58(1)	-19(1)	-5(1)	-21(1)
Ru	56(1)	47(1)	41(1)	-4(1)	-1(1)	-18(1)
O(1W)	237(9)	401(15)	199(8)	-59(9)	49(7)	-178(10)

Table 5. Hydrogen coordinates (x 10^4) and isotropic displacement parameters (A^2 x 10^3) for **12**.

Atom	x	y	z	U(eq)
H(1)	9091	5717	1873	68
H(3)	13141	5934	1779	104
H(4)	14388	4971	871	123
H(5)	13378	4650	-187	133
H(6)	11172	5357	-344	144
H(7)	9900	6297	572	123
H(8)	4435	7576	2048	71
H(9)	2430	8702	2197	82
H(11)	4119	10196	3694	74
H(12)	6015	8991	3531	68
H(13A)	716	10338	2187	134
H(13B)	-126	10674	2892	134
H(13C)	500	9428	2791	134
H(14A)	1810	10795	4077	135
H(14B)	833	11618	3556	135
H(14C)	2373	11573	3491	135
H(17)	4881	8316	5410	84
H(19)	2422	7313	4048	75
H(21A)	7554	7758	4830	127
H(21B)	7779	6463	4906	127
H(21C)	7166	7159	5561	127
H(22A)	2933	8885	6081	189
H(22B)	1707	9765	5791	189
H(22C)	3139	9804	5481	189
H(23A)	961	8832	4345	161
H(23B)	396	9081	5133	161
H(23C)	878	7854	4924	161
H(24A)	4206	5072	3566	105
H(24B)	4733	5845	2963	105
H(24C)	3214	6056	3173	105
H(27)	12594	4169	3028	84
H(29)	10650	2688	1652	90
H(31A)	11524	5225	3948	104
H(31B)	10115	5021	4203	104
H(31C)	10236	6003	3634	104
H(32A)	14550	3598	2150	194
H(32B)	14999	2307	2197	194
H(32C)	14207	2899	2849	194
H(33A)	12630	1503	1559	214
H(33B)	14030	1742	1314	214
H(33C)	12730	2479	981	214
H(34A)	8335	2926	1653	130
H(34B)	7491	3939	2034	130
H(34C)	7879	2791	2472	130
H(36)	7313	7563	864	190
H(37)	6921	8989	11	365
H(39)	7245	11018	1549	131
H(40)	7531	9542	2354	89
H(41A)	6092	12351	729	326
H(41B)	6405	12670	-91	326

H(41C)	7570	12311	469	326
H(42A)	7071	10213	-709	844
H(42B)	6976	11485	-887	844
H(42C)	5687	11022	-681	844
H(84A)	6053	4354	4349	93
H(84B)	7140	4707	4833	93
H(85A)	8722	3686	4218	100
H(85B)	7633	3308	3748	100

Table 6. Torsion angles [deg] for up030.

C(7)-C(2)-C(3)-C(4)	-2.4(9)
S(1)-C(2)-C(3)-C(4)	-179.7(5)
C(2)-C(3)-C(4)-C(5)	0.7(10)
C(3)-C(4)-C(5)-C(6)	1.5(11)
C(4)-C(5)-C(6)-C(7)	-2.1(12)
C(3)-C(2)-C(7)-C(6)	1.8(10)
S(1)-C(2)-C(7)-C(6)	179.0(5)
C(5)-C(6)-C(7)-C(2)	0.5(12)
N(1)-C(8)-C(9)-C(10)	3.1(8)
C(8)-C(9)-C(10)-N(2)	-179.7(5)
C(8)-C(9)-C(10)-C(11)	0.3(7)
N(2)-C(10)-C(11)-C(12)	178.4(5)
C(9)-C(10)-C(11)-C(12)	-1.7(7)
C(10)-C(11)-C(12)-N(1)	-0.1(7)
C(20)-C(15)-C(16)-C(17)	-3.5(7)
N(4)-C(15)-C(16)-C(17)	-172.4(4)
C(20)-C(15)-C(16)-C(21)	177.3(4)
N(4)-C(15)-C(16)-C(21)	8.4(7)
C(15)-C(16)-C(17)-C(18)	1.7(7)
C(21)-C(16)-C(17)-C(18)	-179.1(5)
C(16)-C(17)-C(18)-C(19)	-0.8(7)
C(16)-C(17)-C(18)-N(3)	178.9(5)
C(17)-C(18)-C(19)-C(20)	1.6(7)
N(3)-C(18)-C(19)-C(20)	-178.1(4)
C(18)-C(19)-C(20)-C(15)	-3.4(7)
C(18)-C(19)-C(20)-C(24)	176.3(4)
C(16)-C(15)-C(20)-C(19)	4.3(6)
N(4)-C(15)-C(20)-C(19)	173.5(4)
C(16)-C(15)-C(20)-C(24)	-175.4(4)
N(4)-C(15)-C(20)-C(24)	-6.2(6)
C(30)-C(25)-C(26)-C(27)	2.1(7)
N(5)-C(25)-C(26)-C(27)	172.6(4)
C(30)-C(25)-C(26)-C(31)	-179.1(4)
N(5)-C(25)-C(26)-C(31)	-8.6(6)
C(25)-C(26)-C(27)-C(28)	-1.3(7)
C(31)-C(26)-C(27)-C(28)	179.8(5)
C(26)-C(27)-C(28)-C(29)	1.0(8)
C(26)-C(27)-C(28)-N(6)	-177.3(5)
C(27)-C(28)-C(29)-C(30)	-1.6(8)
N(6)-C(28)-C(29)-C(30)	176.7(5)
C(28)-C(29)-C(30)-C(25)	2.4(8)
C(28)-C(29)-C(30)-C(34)	-174.4(5)
C(26)-C(25)-C(30)-C(29)	-2.6(7)
N(5)-C(25)-C(30)-C(29)	-173.0(4)

C(26)-C(25)-C(30)-C(34)	174.1(4)
N(5)-C(25)-C(30)-C(34)	3.7(7)
N(7)-C(36)-C(37)-C(38)	3(2)
C(36)-C(37)-C(38)-N(8)	177.9(15)
C(36)-C(37)-C(38)-C(39)	2(2)
N(8)-C(38)-C(39)-C(40)	-179.4(12)
C(37)-C(38)-C(39)-C(40)	-3.4(17)
C(38)-C(39)-C(40)-N(7)	0.3(13)
N(4)-C(84)-C(85)-N(5)	-1.4(6)
C(11)-C(12)-N(1)-C(8)	3.1(7)
C(11)-C(12)-N(1)-Ru	171.9(4)
C(9)-C(8)-N(1)-C(12)	-4.6(7)
C(9)-C(8)-N(1)-Ru	-173.7(4)
C(9)-C(10)-N(2)-C(13)	5.3(8)
C(11)-C(10)-N(2)-C(13)	-174.8(5)
C(9)-C(10)-N(2)-C(14)	179.7(5)
C(11)-C(10)-N(2)-C(14)	-0.3(8)
C(17)-C(18)-N(3)-C(22)	2.7(8)
C(19)-C(18)-N(3)-C(22)	-177.6(5)
C(17)-C(18)-N(3)-C(23)	-176.2(5)
C(19)-C(18)-N(3)-C(23)	3.6(8)
N(5)-C(35)-N(4)-C(15)	-173.7(4)
Ru-C(35)-N(4)-C(15)	2.0(7)
N(5)-C(35)-N(4)-C(84)	0.2(5)
Ru-C(35)-N(4)-C(84)	175.9(3)
C(20)-C(15)-N(4)-C(35)	89.0(6)
C(16)-C(15)-N(4)-C(35)	-101.8(5)
C(20)-C(15)-N(4)-C(84)	-84.8(5)
C(16)-C(15)-N(4)-C(84)	84.3(5)
C(85)-C(84)-N(4)-C(35)	0.8(6)
C(85)-C(84)-N(4)-C(15)	175.8(4)
N(4)-C(35)-N(5)-C(25)	-162.0(4)
Ru-C(35)-N(5)-C(25)	22.3(7)
N(4)-C(35)-N(5)-C(85)	-1.2(5)
Ru-C(35)-N(5)-C(85)	-176.9(4)
C(30)-C(25)-N(5)-C(35)	-113.2(5)
C(26)-C(25)-N(5)-C(35)	76.2(6)
C(30)-C(25)-N(5)-C(85)	86.1(6)
C(26)-C(25)-N(5)-C(85)	-84.5(5)
C(84)-C(85)-N(5)-C(35)	1.7(6)
C(84)-C(85)-N(5)-C(25)	165.5(4)
C(29)-C(28)-N(6)-C(33)	0.7(9)
C(27)-C(28)-N(6)-C(33)	179.0(6)
C(29)-C(28)-N(6)-C(32)	-172.2(6)
C(27)-C(28)-N(6)-C(32)	6.0(9)
C(37)-C(36)-N(7)-C(40)	-6.0(15)
C(37)-C(36)-N(7)-Ru	173.0(11)
C(39)-C(40)-N(7)-C(36)	4.5(10)
C(39)-C(40)-N(7)-Ru	-174.4(5)
C(39)-C(38)-N(8)-C(41)	-7(2)
C(37)-C(38)-N(8)-C(41)	177.4(16)
C(39)-C(38)-N(8)-C(42)	178.2(19)
C(37)-C(38)-N(8)-C(42)	3(3)
Ru-C(1)-S(1)-C(2)	-162.7(3)
C(3)-C(2)-S(1)-C(1)	-136.7(5)
C(7)-C(2)-S(1)-C(1)	46.0(6)
S(1)-C(1)-Ru-C(35)	-119.4(3)
S(1)-C(1)-Ru-N(7)	62.4(3)

S(1)-C(1)-Ru-N(1)	78.9(7)
S(1)-C(1)-Ru-Cl(1)	-26.7(3)
S(1)-C(1)-Ru-Cl(2)	153.4(3)
N(4)-C(35)-Ru-C(1)	168.9(4)
N(5)-C(35)-Ru-C(1)	-16.2(4)
N(4)-C(35)-Ru-N(7)	-47(3)
N(5)-C(35)-Ru-N(7)	128(3)
N(4)-C(35)-Ru-N(1)	-16.3(4)
N(5)-C(35)-Ru-N(1)	158.5(4)
N(4)-C(35)-Ru-Cl(1)	75.6(4)
N(5)-C(35)-Ru-Cl(1)	-109.5(4)
N(4)-C(35)-Ru-Cl(2)	-105.0(4)
N(5)-C(35)-Ru-Cl(2)	69.8(4)
C(36)-N(7)-Ru-C(1)	63.4(7)
C(40)-N(7)-Ru-C(1)	-117.8(5)
C(36)-N(7)-Ru-C(35)	-81(3)
C(40)-N(7)-Ru-C(35)	98(3)
C(36)-N(7)-Ru-N(1)	-111.9(7)
C(40)-N(7)-Ru-N(1)	67.0(5)
C(36)-N(7)-Ru-Cl(1)	156.4(6)
C(40)-N(7)-Ru-Cl(1)	-24.7(5)
C(36)-N(7)-Ru-Cl(2)	-22.9(6)
C(40)-N(7)-Ru-Cl(2)	155.9(5)
C(12)-N(1)-Ru-C(1)	-105.8(6)
C(8)-N(1)-Ru-C(1)	62.1(7)
C(12)-N(1)-Ru-C(35)	92.7(4)
C(8)-N(1)-Ru-C(35)	-99.5(3)
C(12)-N(1)-Ru-N(7)	-88.9(3)
C(8)-N(1)-Ru-N(7)	78.9(3)
C(12)-N(1)-Ru-Cl(1)	0.0(3)
C(8)-N(1)-Ru-Cl(1)	167.8(3)
C(12)-N(1)-Ru-Cl(2)	-179.9(3)
C(8)-N(1)-Ru-Cl(2)	-12.1(3)
