# Supplementary material for: Hexacoordinate Ru-based olefin metathesis catalysts with pH-responsive N-heterocyclic carbene (NHC) and N-donor ligands for ROMP reactions in non-aqueous, aqueous and emulsion conditions
Source: Beilstein J Org Chem. 2015 Oct 21;11:1960–72. doi: 10.3762/bjoc.11.212 (PMC4660970; doi:10.3762/bjoc.11.212)

Supporting Information

for

Hexacoordinate Ru-based olefin metathesis catalysts with pH-responsive N-heterocyclic carbene (NHC) and N-donor ligands for ROMP reactions in non-aqueous, aqueous and emulsion conditions

Shawna L. Balof [1], K. Owen Nix[2], Matthew S. Olliff [2], Sarah E. Roessler [2], Arpita Saha[2], Kevin B. Müller [3], Ulrich Behrens [4], Edward J. Valente[5] and Hans-Jörg Schanz[2]*

Address: [1]Department of Chemistry & Biochemistry, The University of Southern Mississippi, 118 College Drive, Hattiesburg, MS 39406-5043, USA, [2]Department of Chemistry, Georgia Southern University, 521 College of Education Drive, Statesboro, GA 30458-8064, USA, [3]BASF SE, G-PM/PD - F206, 67056 Ludwigshafen, Germany, [4]BASF SE, Basic Chemicals Research, GCB/C – M313, 67056 Ludwigshafen, Germany and [5]Department of Chemistry, University of Portland, 5000 N. Willamette Blvd., Portland, OR 97203, USA

*Corresponding author

Email: Hans-Jörg Schanz - hschanz@georgiasouthern.edu

^{1}H, ^{13}C and ^{31}P NMR spectra of the synthesized Ru-complexes 9, 11 and 12 as well as kinetic experimental data

NMR-Spectroscopy (20 °C unless stated otherwise)

Complex 9

^{1}H NMR

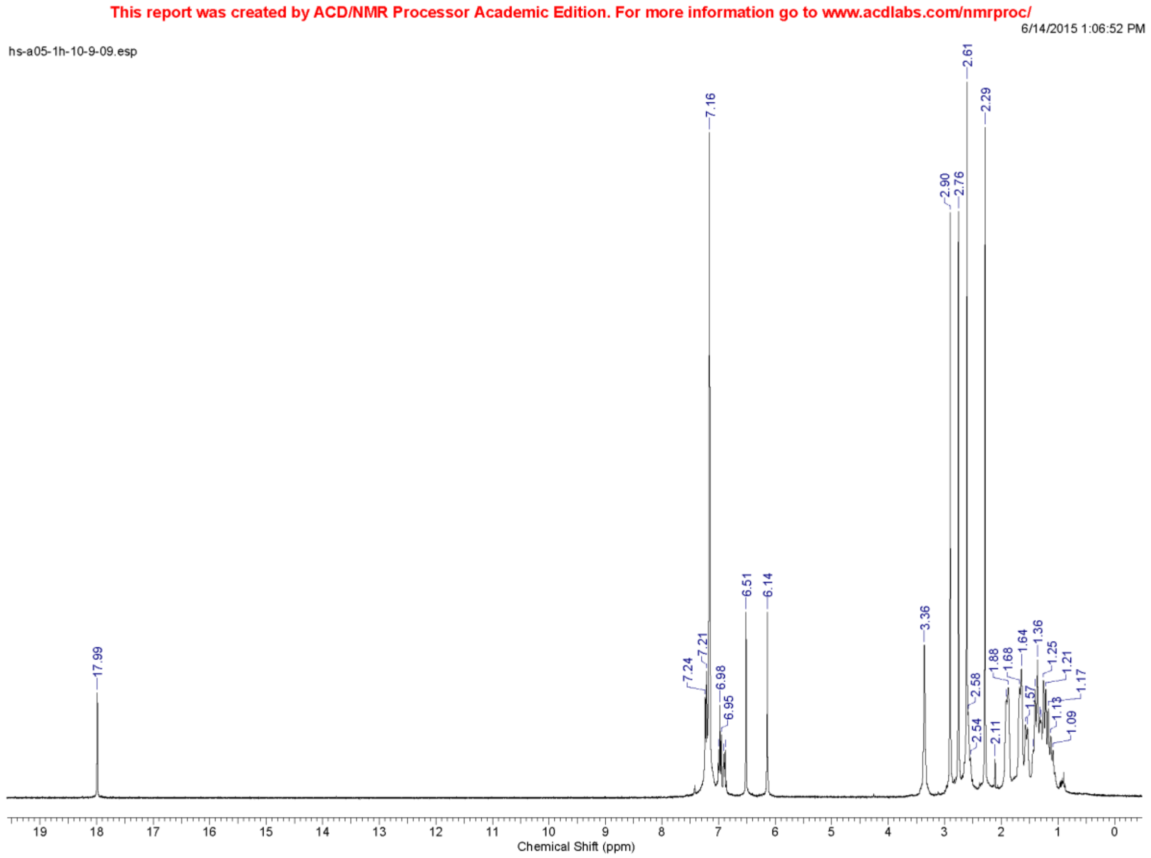

^{13}C NMR

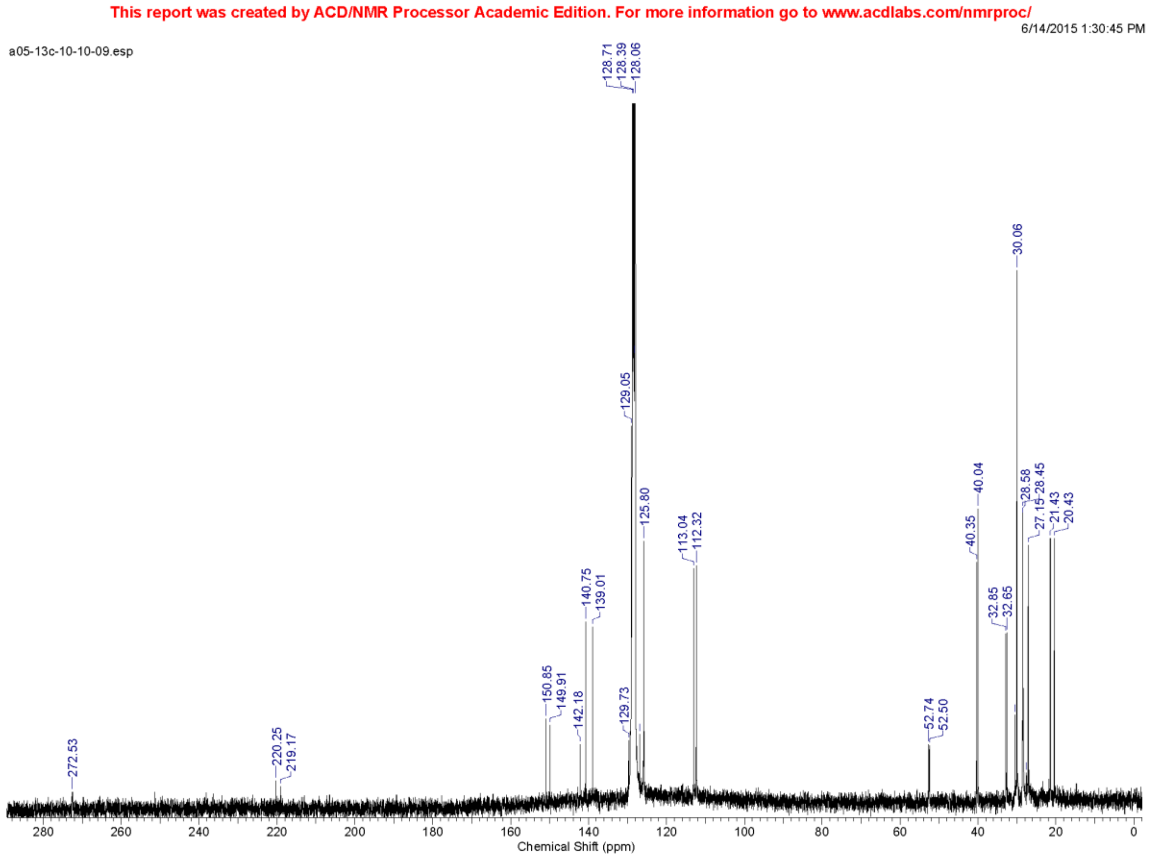

This report was created by ACD/NMR Processor Academic Edition. For more information go to www.acdlabs.com/nmrproc/
6/14/2015 1:30:45 PM
a05-13c-10-10-09.esp
128.71
128.39
128.06
30.06
129.05
40.04
28.58
28.45
125.80
27.15
21.43
20.43
40.35
113.04
112.32
140.75
139.01
32.85
32.65
150.85
149.91
142.18
129.73
52.74
52.50
272.53
220.25
219.17
Chemical Shift (ppm)

Expanded Spectra:

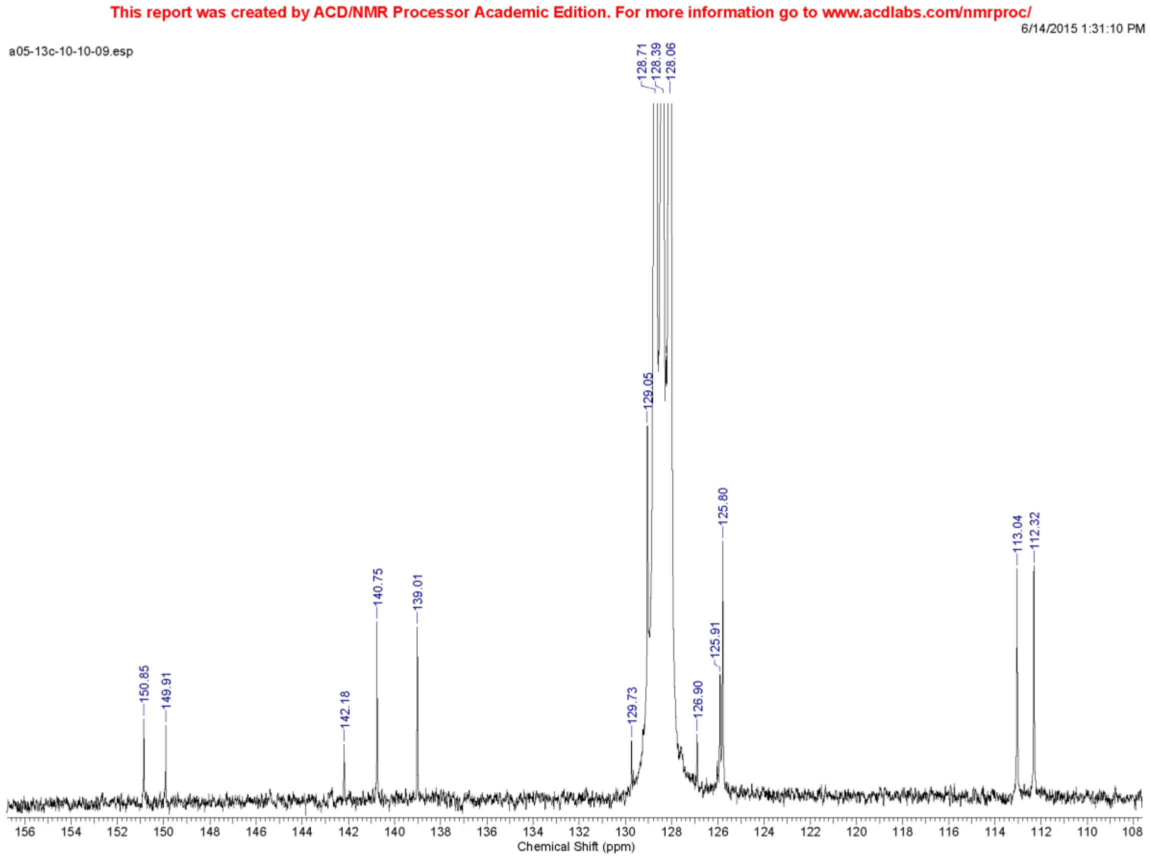

This report was created by ACD/NMR Processor Academic Edition. For more information go to www.acdlabs.com/nmrproc/
6/14/2015 1:31:53 PM

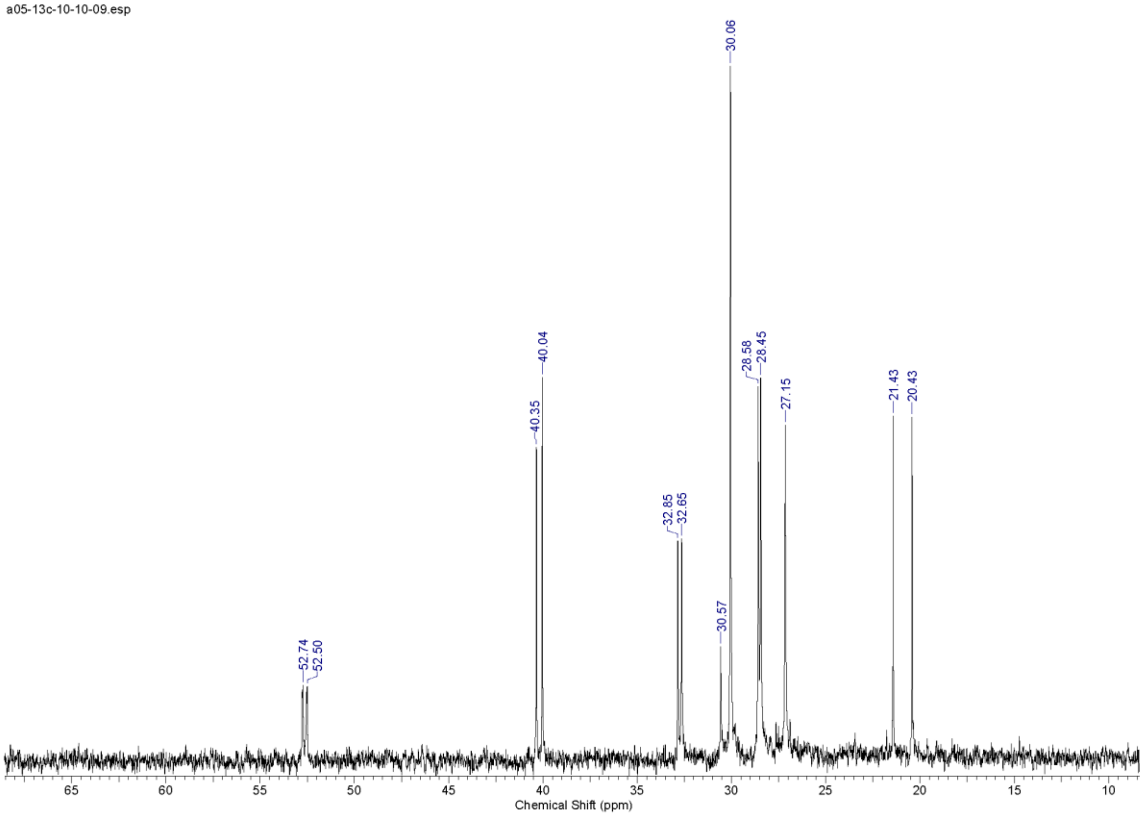

^{31}P NMR

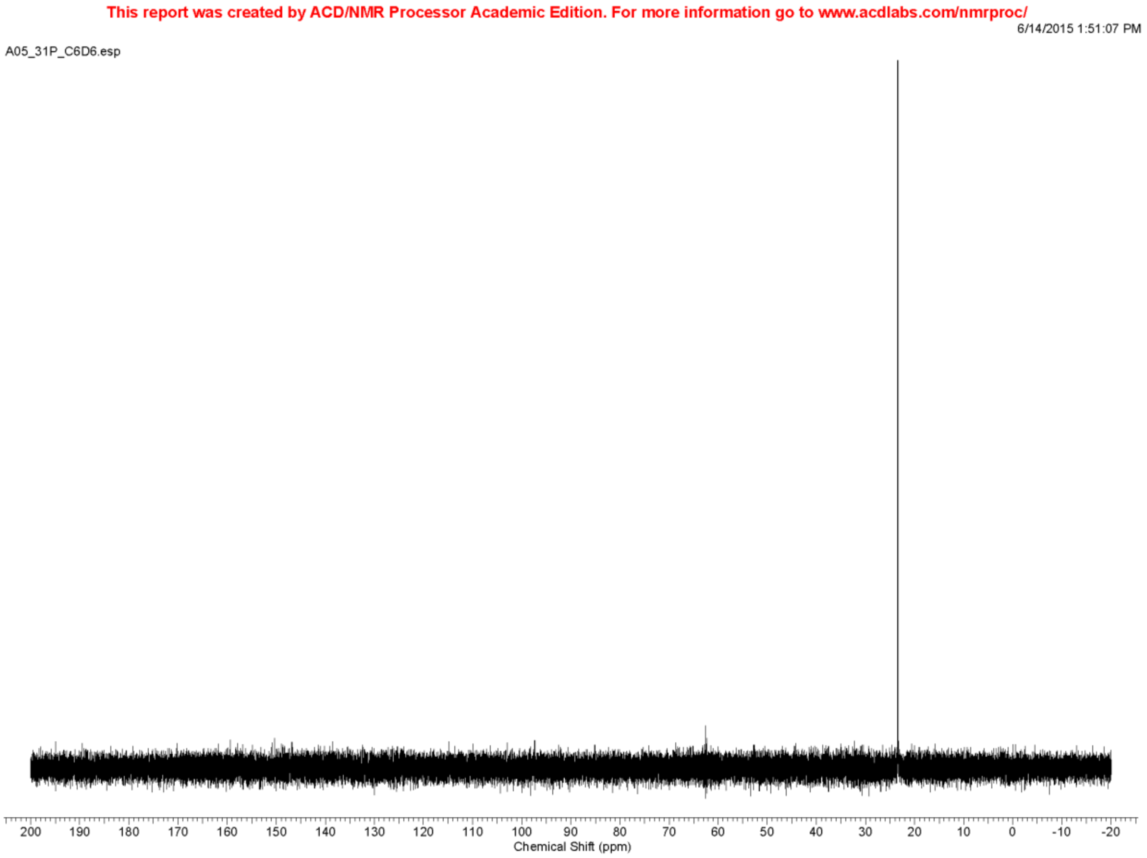
This report was created by ACD/NMR Processor Academic Edition. For more information go to www.acdlabs.com/nmrproc/
6/14/2015 1:51:07 PM
A05_31P_C6D6.esp
200 190 180 170 160 150 140 130 120 110 100 90 80 70 60 50 40 30 20 10 0 -10 -20
Chemical Shift (ppm)

Complex 11

^1H NMR

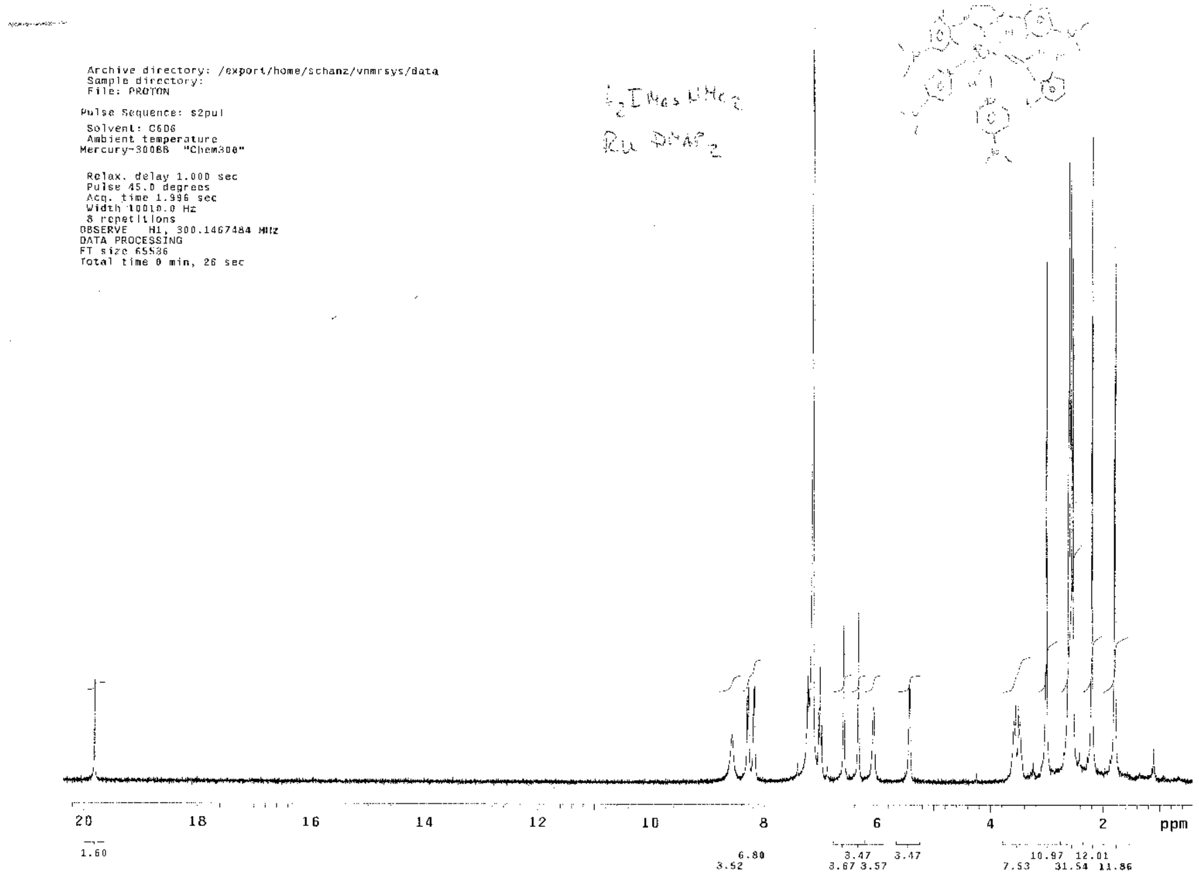

^{13}C NMR

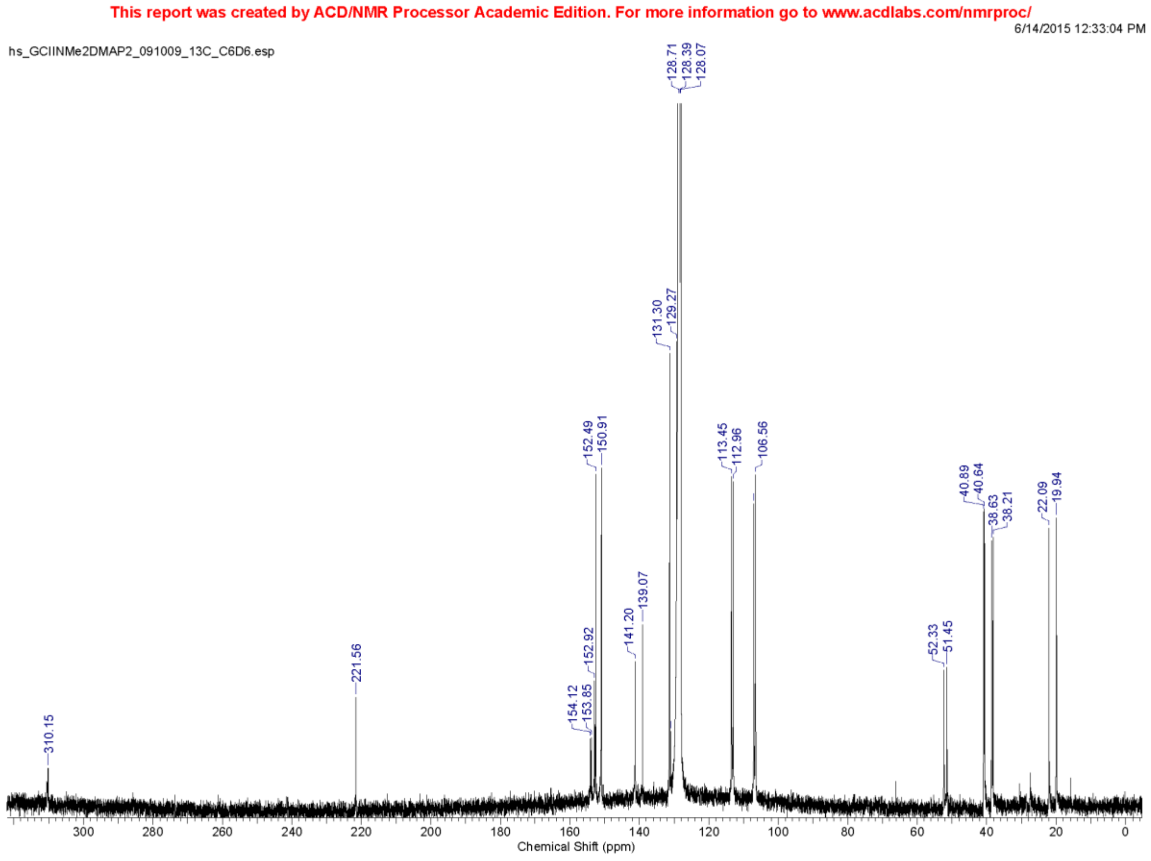
This report was created by ACD/NMR Processor Academic Edition. For more information go to www.acdlabs.com/nmrproc/
6/14/2015 12:33:04 PM
hs_GCIINMe2DMAP2_091009_13C_C6D6.esp
310.15
221.56
154.12
153.85
152.92
152.49
150.91
141.20
139.07
131.30
129.27
128.71
128.39
128.07
113.45
112.96
106.56
52.33
51.45
40.89
40.64
38.63
38.21
22.09
19.94
300 280 260 240 220 200 180 160 140 120 100 80 60 40 20 0
Chemical Shift (ppm)

Complex 12

^{1}H NMR

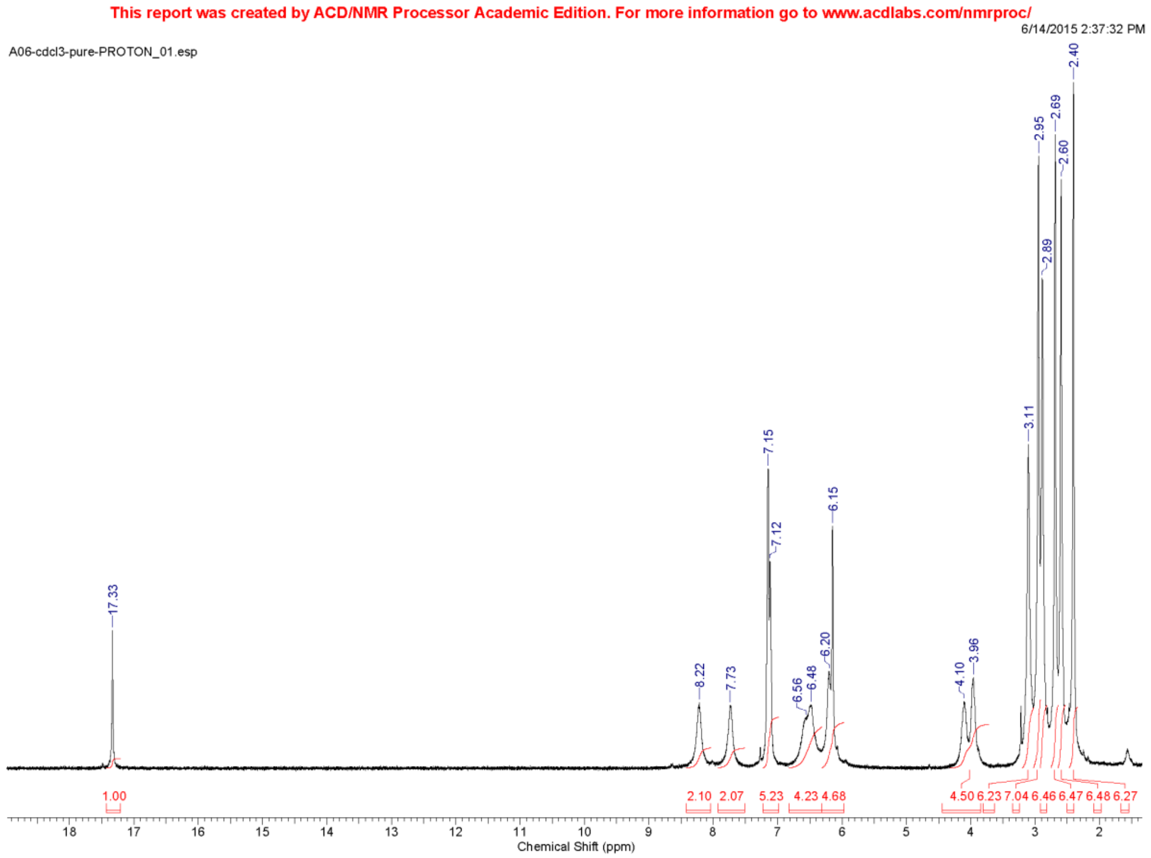

^{1}H NMR (-20 °C)

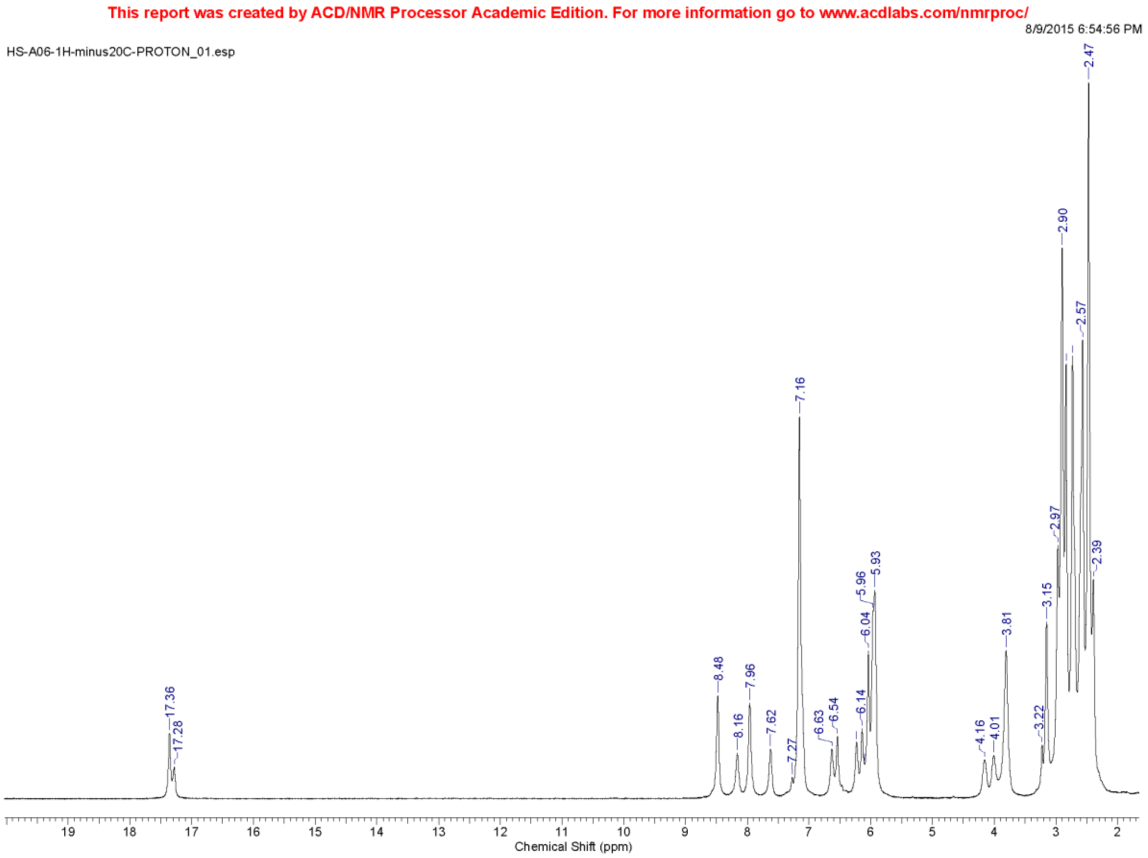

This report was created by ACD/NMR Processor Academic Edition. For more information go to www.acdlabs.com/nmrproc/
8/9/2015 6:54:56 PM
HS-A06-1H-minus20C-PROTON_01.esp
17.36
17.28
8.48
8.16
7.96
7.62
7.27
7.16
6.63
6.54
6.14
6.04
5.96
5.93
4.16
4.01
3.81
3.22
3.15
2.97
2.90
2.57
2.47
2.39
19
18
17
16
15
14
13
12
11
10
9
8
7
6
5
4
3
2
Chemical Shift (ppm)

^{13}C NMR

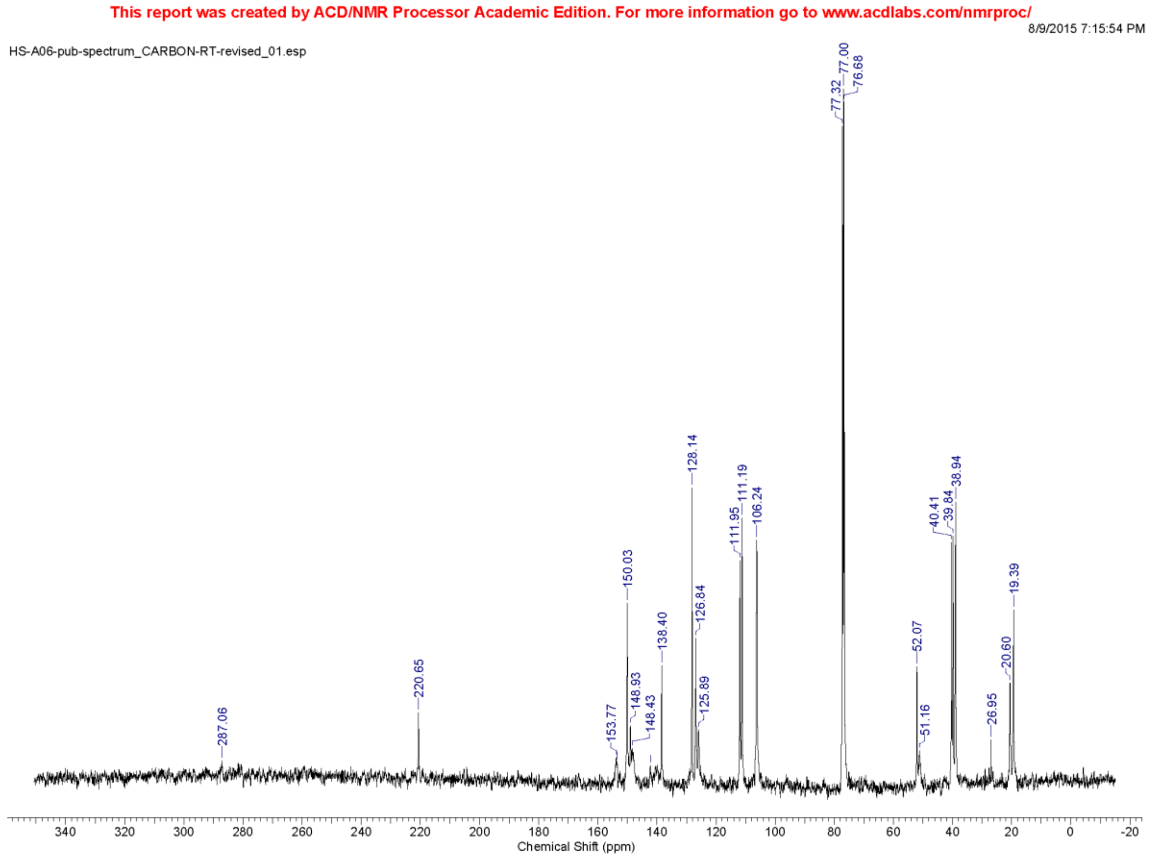

This report was created by ACD/NMR Processor Academic Edition. For more information go to www.acdlabs.com/nmrproc/
8/9/2015 7:15:54 PM
HS-A06-pub-spectrum_CARBON-RT-revised_01.esp
287.06
220.65
153.77
150.03
148.93
148.43
138.40
128.14
126.84
125.89
111.95
111.19
106.24
77.32
77.00
76.68
52.07
51.16
40.41
39.84
38.94
26.95
20.60
19.39
Chemical Shift (ppm)

^{13}C NMR (-20 °C)

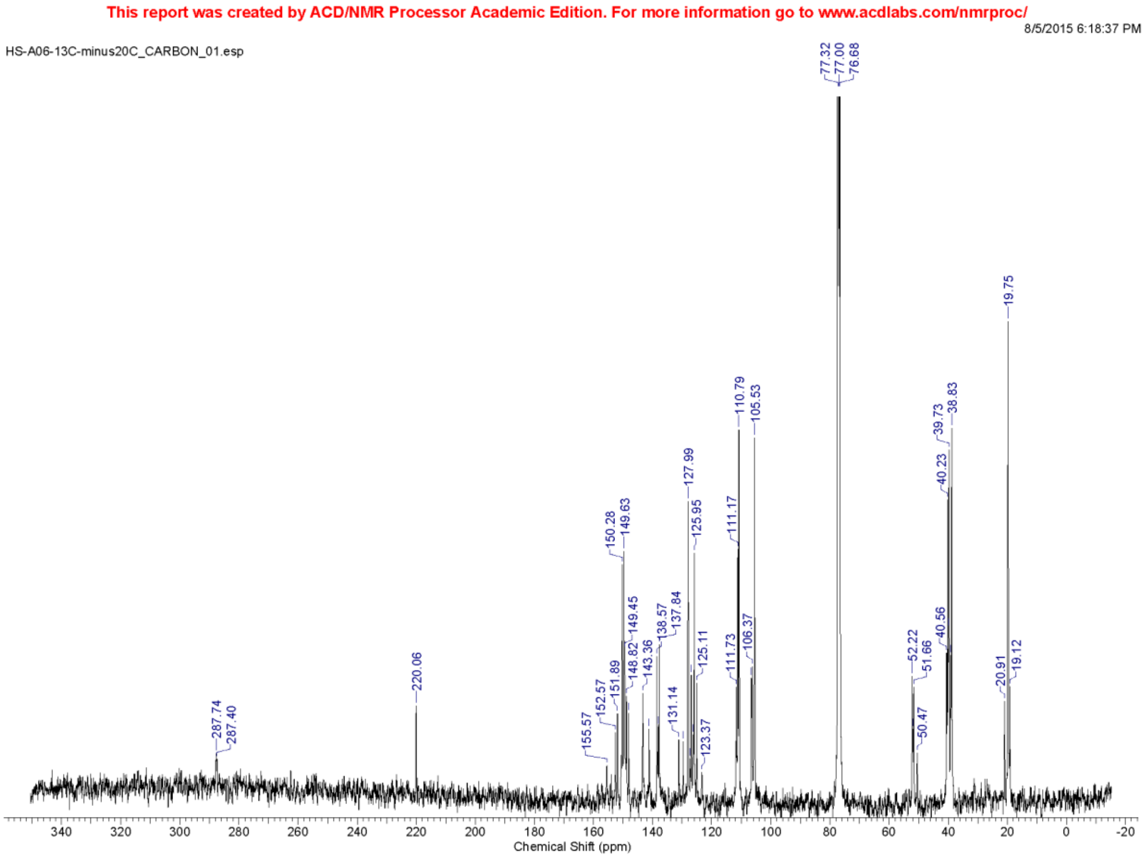

This report was created by ACD/NMR Processor Academic Edition. For more information go to www.acdlabs.com/nmrproc/
8/5/2015 6:18:37 PM
HS-A06-13C-minus20C_CARBON_01.esp
77.32
77.00
76.68
19.75
110.79
105.53
39.73
38.83
40.23
127.99
111.17
125.95
150.28
149.63
149.45
138.57
137.84
106.37
40.56
52.22
51.66
111.73
125.11
148.82
143.36
220.06
151.89
152.57
20.91
19.12
131.14
287.74
287.40
155.57
50.47
123.37
Chemical Shift (ppm)

Expanded Spectra

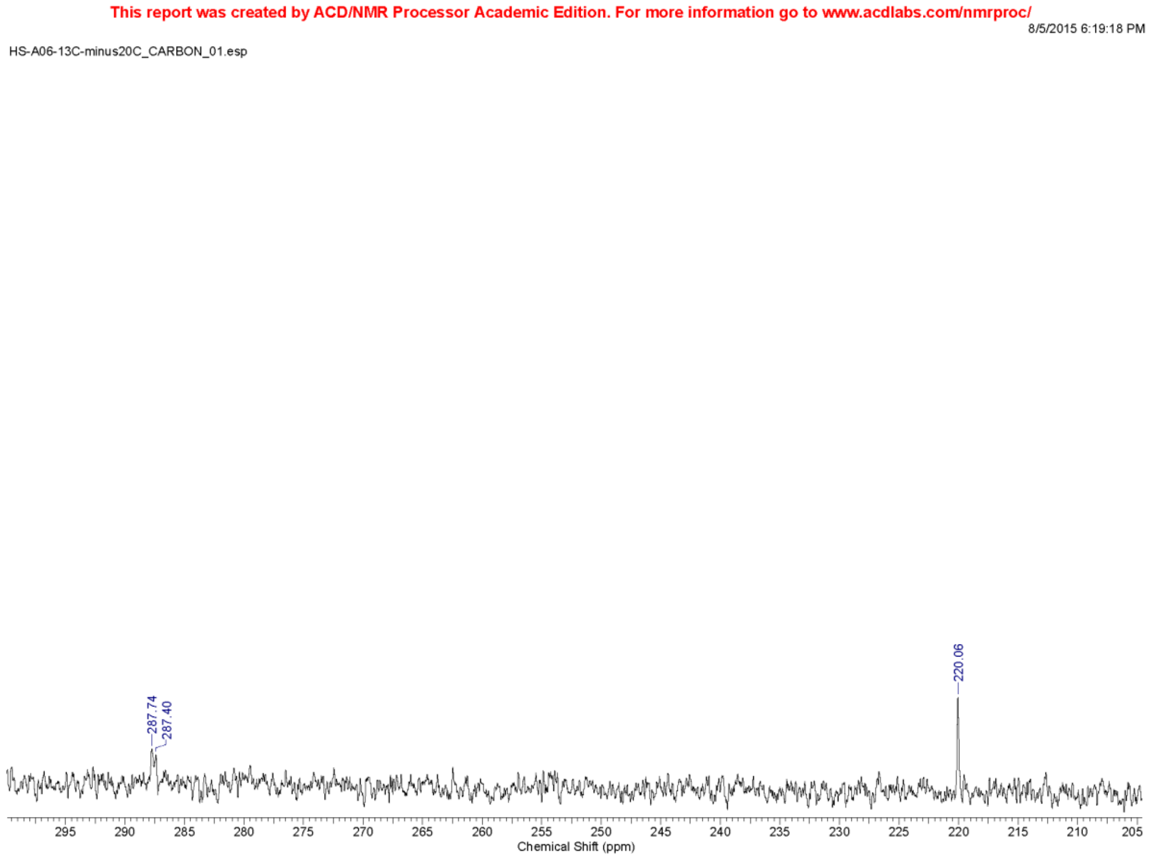

This report was created by ACD/NMR Processor Academic Edition. For more information go to www.acdlabs.com/nmrproc/

8/5/2015 6:20:10 PM

HS-A06-13C-minus20C_CARBON_01.esp

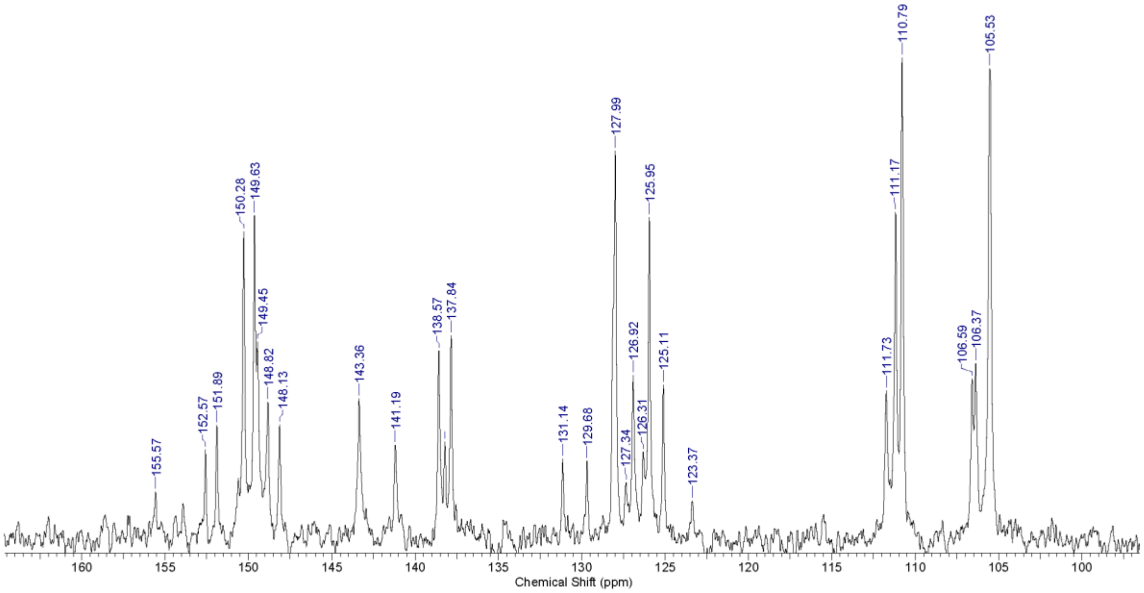

This report was created by ACD/NMR Processor Academic Edition. For more information go to www.acdlabs.com/nmrproc/

8/5/2015 6:20:35 PM

HS-A06-13C-minus20C_CARBON_01.esp

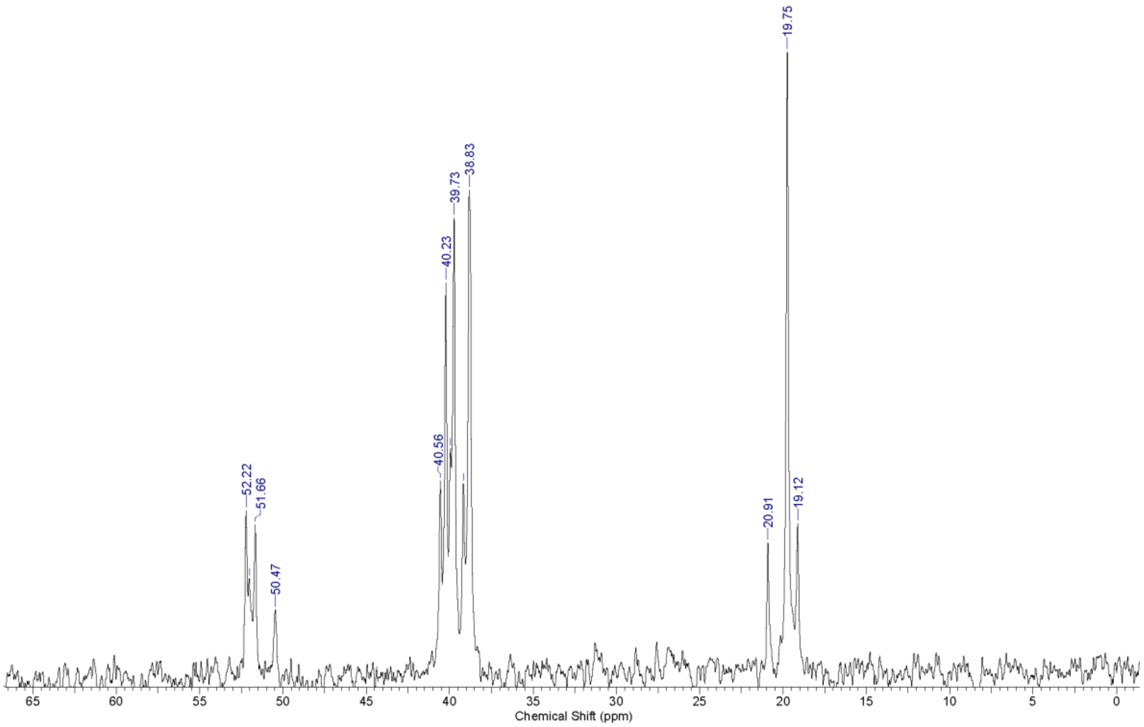

Kinetic Data

ROMP of COE

Catalyst **9**

Room Temperature
0.8% conversion after 1 hour

At 60 degrees C

t (min.)	% conversion
0	0
1	0
2	0
4	11.89
6	30.56
8	50.13
10	68.6
12	79.64
14	86.1
16	90.18
18	92.85
20	94.84
22	95.42
24	96.09

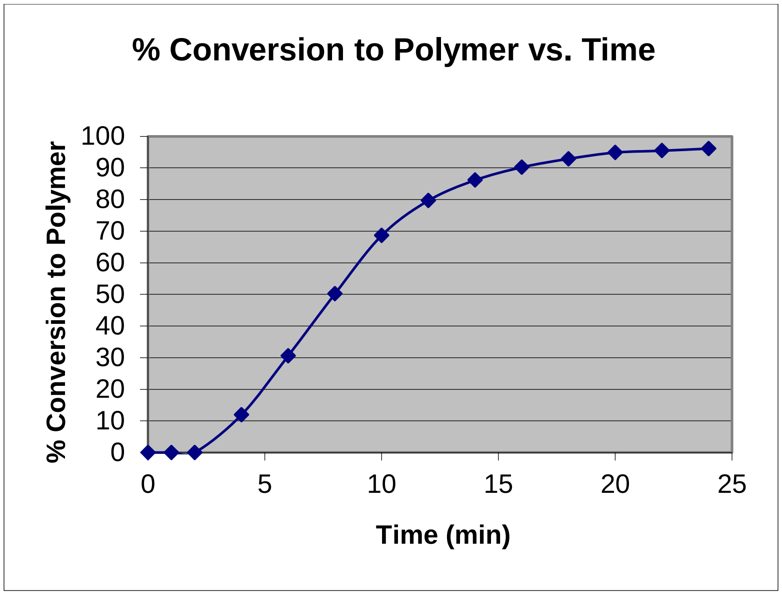

Catalyst **11**

DMAP catalyst (no acid)

t (min)	% Conversion to Polymer
0	0
4	39.25
8	60.89
10	71.33
13	80.99
16	90.17
19	93.43

DMAP catalyst (+ 2 equiv H_3PO_4)

T (min)	% Conversion to Polymer
0	0
1	28.62
3	54
6	70.72
9	82.99
12	90.57
15	96.84

DMAP catalyst (+ 2 equiv H_3PO_4)

t (min)	% Conversion to Polymer
0	0
1	11.33
3	21.21
6	27.98
9	32.37
12	35.8
30	40.76

no change after 30 min.

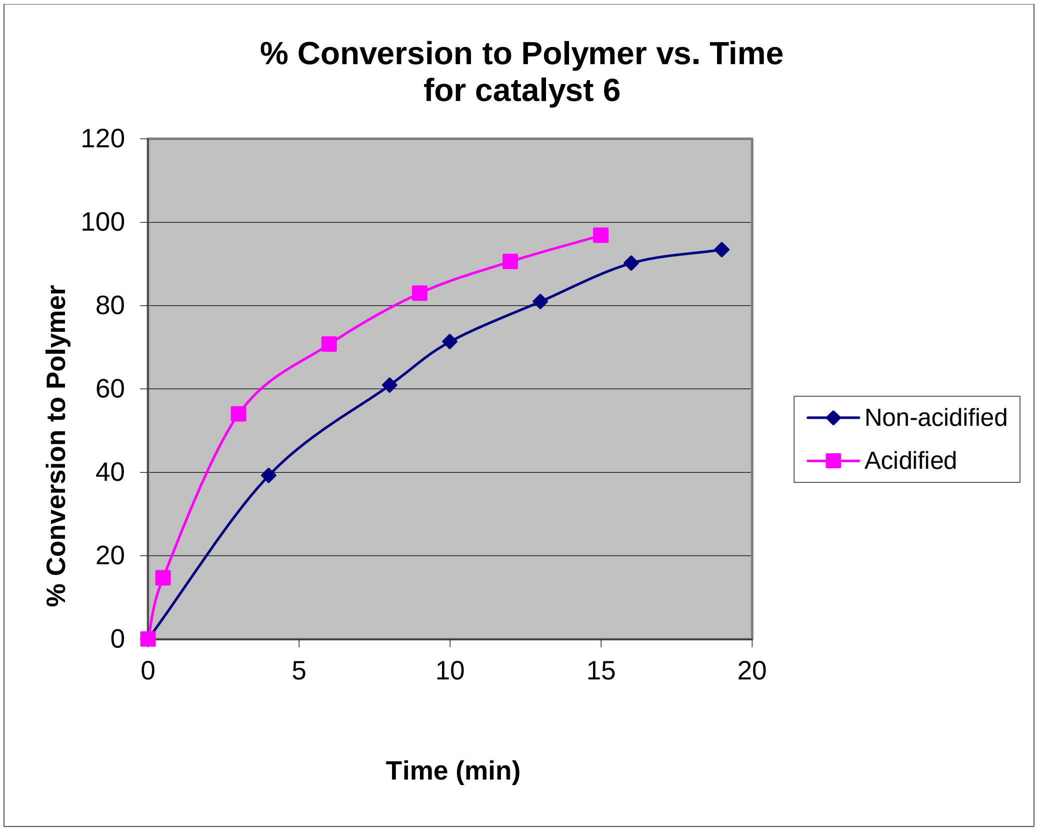

Catalyst **12**

Room Temperature

No acid-- 3.91% conversion after 1 hour

with 2 equiv H3PO4 0.9% conversion after 1 hour

At 60 degrees C

t (min.)	% Conversion
0	0
1	0
2	1.21
4	3.89
6	6.12
8	9.18
10	12.86
12	16.73

14	21.58
16	25.07
18	27.15
20	28.78
22	29.84
24	30.69
26	31.28
28	31.81
30	32.4
32	32.81
34	33.29
36	33.75
38	33.97
40	34.22
42	34.56
44	34.71
46	34.98
48	35.14
50	35.33
52	35.54
54	35.79
56	35.91
58	36.17
60	36.34
120	37.78
180	no change

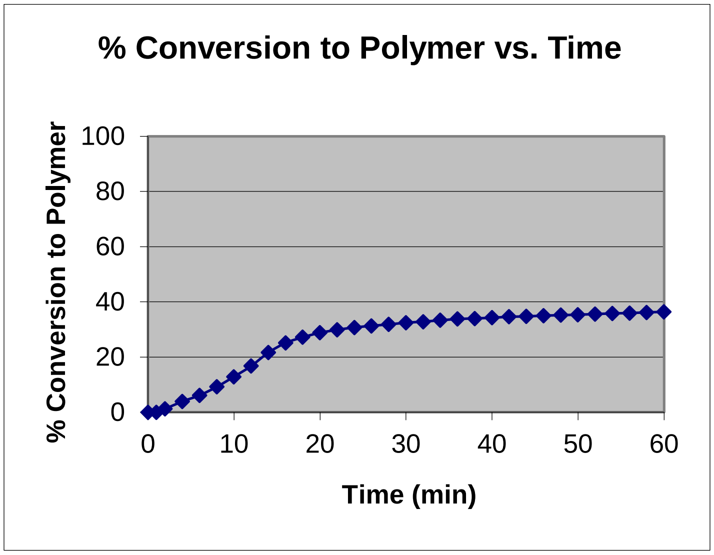

RCM of DEDAM

Catalyst **10**

RCM of DEDAM 1.0 mM solution in C_6D_6
1.0% catalyst loading

Room Temperature			At 60 degrees C	
t (min.)	% Conversion		T (min.)	% Conversion
0	0		0	0
1	0		1	7.23
2	0.12		2	19.9
4	0.2		4	36.72
6	0.27		6	44.05
8	0.37		8	49.64
10	0.42		10	52.6
12	0.45		12	57.04
14	0.56		14	58.87
16	0.63		16	66.73
18	0.7		18	70.46
20	0.78		20	72.67
22	0.81		22	74.35
24	0.87		24	75.86
26	0.93		26	76.48
28	1.1		28	78.23
30	1.19		30	80.79
32	1.24		32	81.76
34	1.29		34	82.33
36	1.38		36	83.39
38	1.43		38	83.59
40	1.53		40	84.62
42	1.58		42	85.05
44	1.72		44	85.67
46	1.83		46	86.15
48	1.89		48	86.68
50	1.93		50	87.12
52	2		52	87.59
54	2.06		54	88.01
56	2.13		56	88.43
58	2.24		58	88.98
60	2.27		60	89.43
			120	93.98

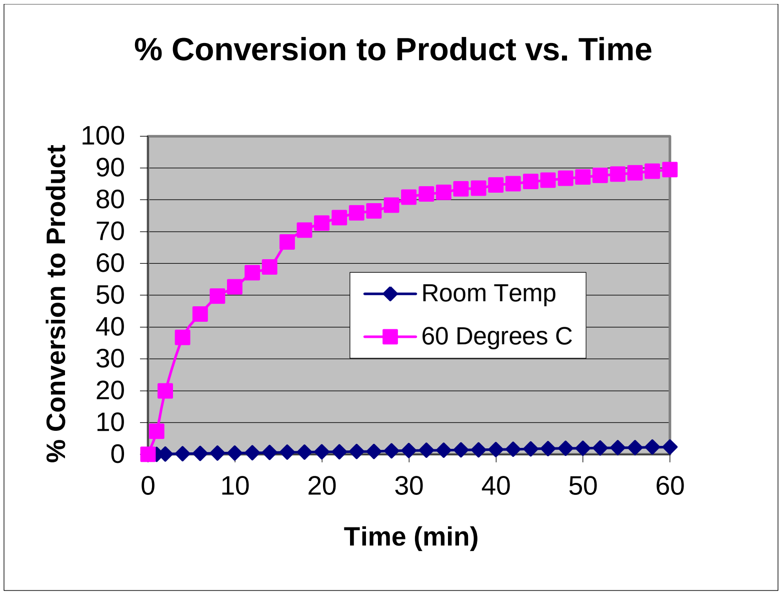

Catalyst **11**

RCM of DEDAM	1.0 mM solution in C6D6
	1.0% catalyst loading

<u>Room Temperature</u>

No acid-- 7.2% conversion after 0.5 hours

<u>with 2 equiv H_3PO_4</u>

t (min.)	% Conversion
0	0
1	2.55
2	7.90
4	18.90
6	26.56
8	34.44
10	38.11
12	40.23
14	42.32
16	43.98
18	44.15
20	44.76
30	46.67
60	47.43
120	47.35

with 4 equiv
H_3PO_4

t (min.)	% Conversion
0	0
1	1.85
2	4.66
4	7.21
6	8.31
8	8.93
10	9.15
30	13.76
60	No change

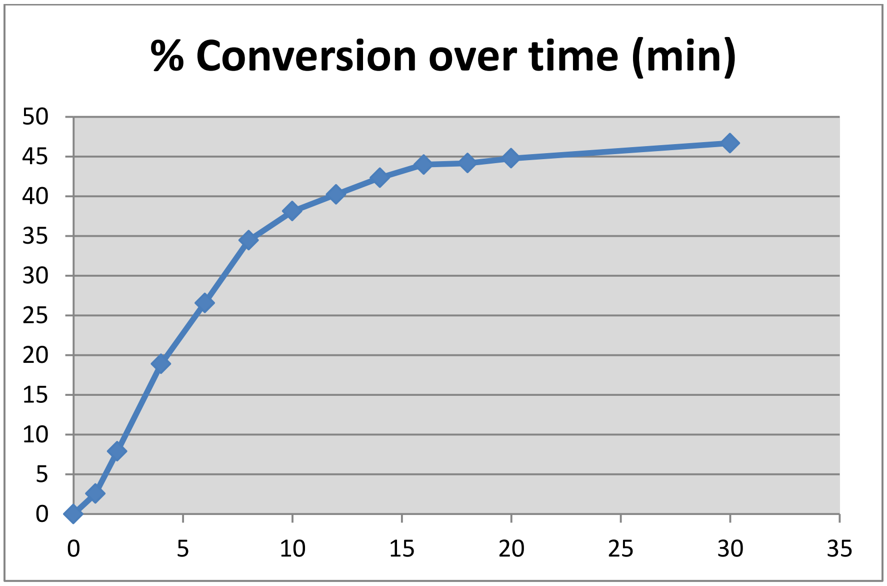

Catalyst **12**

RCM of DEDAM

1.0 mM solution in C6D6
1.0% catalyst loading

Room Temperature
No acid-- 1.22% conversion after 1 hour

At 60 degrees C

t (min.)	% Conversion
0	0
1	1.27
2	5.43

4	10.19
6	18.53
8	24.68
10	30.25
12	33.19
14	37.42
16	39.64
18	41.68
20	43.4
22	45.16
24	46.69
26	47.53
28	48.62
30	49.67
32	50.53
34	51.52
36	52.39
38	53.11
40	53.98
42	54.66
44	54.91
46	55.15
48	55.83
50	56.11
52	56.46
54	56.79
56	56.98
58	57.02
60	57.23
120	60.21
180	61.09
240	no change

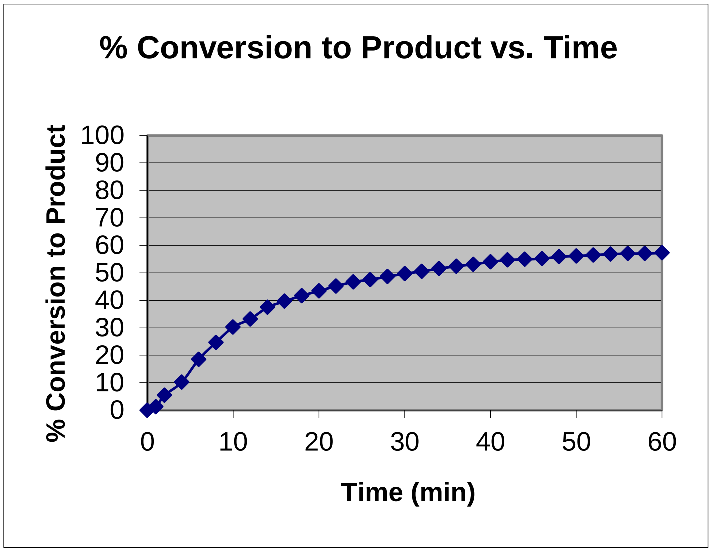

Supplement: File 2 — 1H, 13C and 31P NMR spectra of the synthesized Ru-complexes 9, 11 and 12 as well as kinetic experimental data. [file Beilstein_J_Org_Chem-11-1960-s002.pdf]
